# Multiclass characterization of frontotemporal dementia variants via multimodal brain network computational inference

**DOI:** 10.1162/netn_a_00285

**Published:** 2023-01-01

**Authors:** Raul Gonzalez-Gomez, Agustín Ibañez, Sebastian Moguilner

**Affiliations:** Latin American Brain Health Institute (BrainLat), Universidad Adolfo Ibañez, Santiago de Chile, Chile; Center for Social and Cognitive Neuroscience, School of Psychology, Universidad Adolfo Ibañez, Santiago de Chile, Chile; Cognitive Neuroscience Center, Universidad de San Andres, Buenos Aires, Argentina; Global Brain Health Institute, University of California San Francisco, San Francisco, CA, USA; Trinity College Dublin, Dublin, Ireland; Department of Neurology, Massachusetts General Hospital and Harvard Medical School, Boston, MA, USA

**Keywords:** Multiclass classification, FTD, FTD variants, Connectivity, Machine learning

## Abstract

Characterizing a particular neurodegenerative condition against others possible diseases remains a challenge along clinical, biomarker, and neuroscientific levels. This is the particular case of frontotemporal dementia (FTD) variants, where their specific characterization requires high levels of expertise and multidisciplinary teams to subtly distinguish among similar physiopathological processes. Here, we used a computational approach of multimodal brain networks to address simultaneous multiclass classification of 298 subjects (one group against all others), including five FTD variants: behavioral variant FTD, corticobasal syndrome, nonfluent variant primary progressive aphasia, progressive supranuclear palsy, and semantic variant primary progressive aphasia, with healthy controls. Fourteen machine learning classifiers were trained with functional and structural connectivity metrics calculated through different methods. Due to the large number of variables, dimensionality was reduced, employing statistical comparisons and progressive elimination to assess feature stability under nested cross-validation. The machine learning performance was measured through the area under the receiver operating characteristic curves, reaching 0.81 on average, with a standard deviation of 0.09. Furthermore, the contributions of demographic and cognitive data were also assessed via multifeatured classifiers. An accurate simultaneous multiclass classification of each FTD variant against other variants and controls was obtained based on the selection of an optimum set of features. The classifiers incorporating the brain’s network and cognitive assessment increased performance metrics. Multimodal classifiers evidenced specific variants’ compromise, across modalities and methods through feature importance analysis. If replicated and validated, this approach may help to support clinical decision tools aimed to detect specific affectations in the context of overlapping diseases.

## Author Summary

The distinction of a neurodegenerative condition against multiple related diseases simultaneously, with overlapping features and high levels of heterogeneity in behavioral, clinical, and neuropathological markers, remains a challenge. Here, we combined structural and functional connectivity markers with diverse methods including graph theory and cognitive markers to perform a multiclass classification of five FTD variants. An optimum set of features was obtained through progressive feature elimination by removing redundant and uninformative variables. Our results for the simultaneous multiclass categorization of each FTD variant and healthy controls achieved a performance up to an area under the curve of 0.95. This approach can help develop clinical decision support tools to detect specific affectations in the context of overlapping neurodegenerative diseases.

## INTRODUCTION

Distinguishing a single neurodegenerative condition against multiple related diseases simultaneously remains a challenge at clinical, biomarker, and neuroscientific levels. In particular, the clinical diagnosis of each frontotemporal dementia (FTD) variant requires high levels of expertise and multidisciplinary teams to distinguish among subtle phenotypes with similar underlying physiopathological processes (Bejanin et al., 2020; Staffaroni et al., 2019; Zetterberg et al., 2019). FTD presents with high levels of heterogeneity in behavioral, clinical, and neuropathological markers (Boeve et al., 2022; Peet et al., 2021; Rohan et al., 2019). Furthermore, biomarkers have been shown to be less sensitive to multiclass differentiation (i.e., classifying one condition against multiple other variants) across neurodegenerative diseases (Moral-Rubio et al., 2021; Tahmasian et al., 2016). Standard positron emission tomography (PET) biomarkers are relatively specific for Alzheimer’s disease (Ossenkoppele et al., 2021), but there are caveats when using PET to distinguish between FTD variants (Tsai et al., 2019). Plasma markers are promising for determining some FTD variants but are not yet massively accessible. Finally, the field of cognitive neuroscience has developed multiple metrics for use with machine learning (ML) classification of neurodegenerative conditions (Bachli et al., 2020; Ibañez et al., 2021a, 2021b; Moguilner et al., 2021), including some FTD variants in particular (Feis et al., 2018; Moral-Rubio et al., 2021; Premi et al., 2016). However, in most cases, the classification is based on binary comparisons without assessing the clinical classification of one condition compared to numerous other conditions. These studies have also usually been performed with small sample comparisons and generally consider unimodal brain features. Thus, a multiclass characterization across FTD variants remains scarce despite different approaches.

Robust, scalable, and affordable biomarkers segregating not only healthy status from disease but also among multiple variants of similar conditions could be assessed via brain network approaches. Functional and structural connectivity based on magnetic resonance imaging (MRI) can be relevant regarding molecular mechanisms, pathological alterations, and clinical symptoms (Meeter et al., 2017; Pievani et al., 2011). Previous research has demonstrated its usefulness in identifying neurodegenerative disorders (Hafkemeijer et al., 2017; Hohenfeld et al., 2018; Jalilianhasanpour et al., 2019) and FTD variants (Chen et al., 2020; Reyes et al., 2018; Whitwell, 2019). However, the currently available research has several limitations. Within-subject variability is usually excluded (Finn et al., 2015; Salvatore et al., 2014). The results are typically biased by unsystematic research, including different modalities (i.e., structural vs. functional connectivity) and methods (i.e., voxel connectivity, region of interest (ROI) analysis, graph theory). Most importantly, comprehensive frameworks integrating different connectivity metrics to simultaneously distinguish between multiple FTD variants have not yet been developed. Thus, a more systematic computational framework that incorporates different connectivity modalities and methods to characterize each FTD variant against multiple other variants and controls has not been developed.

Recent artificial intelligence developments for multiclass classification (Churcher et al., 2021; Gao et al., 2021; Hou et al., 2021) using ML are well suited to combine different connectivity modalities and methods to test the power of multiclass classification. The ML framework requires minimal assumptions and is more robust than parametric approaches regarding data heterogeneity (Makridakis et al., 2018; Poldrack et al., 2019). Classification can be performed with many variables with nonlinear interactions (Bzdok et al., 2018; Bzdok et al., 2017). Combining ML with progressive feature elimination can identify the main predictors, enhance classification, and provide a top assortment of features to classify outcomes (Montavon et al., 2018; Nicholls et al., 2020). Moreover, these methods can account for additional sources of heterogeneity, such as demographic and cognitive measures, in combination with brain network features.

The general aim of this study was to optimize the number of multimodal sources of information and to develop an effective multifeatured and multiclass classification of FTD variants. To reduce dimensionality due to the large number of variables (i.e., voxel-wise information adding up to the scale of 10^6^ variables), group-level statistical analyses were employed before data-driven progressive elimination to overcome computational constraints. These statistical approaches enabled comparing our findings with previous studies in FTD variants (Agosta et al., 2012; Bharti et al., 2017; Iaccarino et al., 2015; Popal et al., 2020; Tovar-Moll et al., 2014; Upadhyay et al., 2016; Whitwell et al., 2009). Moreover, by complementing the statistical approaches with a subsequent ML classification, we followed hybrid methodologies reported in the literature (Dottori et al., 2017; Fittipaldi et al., 2020; Gonzalez Campo et al., 2020; Kassraian-Fard et al., 2016; McMillan et al., 2012). In total, 298 subjects were analyzed having different FTD variants, namely, behavioral variant FTD (bvFTD; *n* = 47), corticobasal syndrome (CBS; *n* = 38), nonfluent variant primary progressive aphasia (nfvPPA; *n* = 34), progressive supranuclear palsy (PSP; *n* = 42), and semantic variant primary progressive aphasia (svPPA; *n* = 38), and healthy controls (HC; *n* = 99). The ML classifier employed was the eXtreme Gradient Boosting (XGBoost) algorithm (Kaufmann et al., 2019), which was used to classify one group against each of the remaining others. Brain network features included two modalities (functional and structural) and three methods (voxel level or raw connectivity, ROI-to-ROI, and graphs). In addition, we incorporated basic demographics (sex, age, years of education) and cognitive measures (disease severity and cognitive screening) into a multifeatured classification. Three hypotheses were advanced: (1) feature optimization will enable accurate simultaneous multiclass classification of each FTD variant relative to other variants and controls, (2) use of cognitive measures in conjunction with connectivity will increase classification accuracy, and (3) the model comprising all modalities and methods will outperform the models with single methods and modalities. By testing these hypotheses, we aimed to assess the robustness of a multiclass computational framework for characterizing each FTD variant simultaneously against all other variants and controls.

## MATERIALS AND METHODS

### Subjects

All the data were obtained from LONI’s databases (https://ida.loni.usc.edu), namely, Neuroimaging in Frontotemporal Dementia (NIFD) and the 4 Repeat Tauopathy Neuroimaging Initiative (4RTNI), both of which are part of the frontotemporal lobar degeneration neuroimaging initiative (https://4rtni-ftldni.ini.usc.edu). Clinical diagnosis of the FTD variants was based on current criteria (Armstrong et al., 2013; Gorno-Tempini et al., 2011; Rascovsky & Grossman, 2013; Rascovsky et al., 2011). Patients did not present any vascular, psychiatric, or other neurological disorders. The inclusion of healthy subjects required confirmation of normal cognitive function, the absence of any disease, and a brain MRI free of lesions or significant white matter changes.

In total, data from 298 subjects were analyzed, including HCs (*n* = 99) and individuals with one of five FTD variants: bvFTD (*n* = 47), CBS (*n* = 38), nfvPPA (*n* = 34), PSP (*n* = 42), and svPPA (*n* = 38). These variants constitute the core FTD spectrum disorders (Olney et al., 2017), and their typical atrophy patterns (Boxer et al., 2006; Lu et al., 2013; Seeley et al., 2009; Whitwell et al., 2010b; Whitwell et al., 2009) were confirmed via voxel-based morphometry (Supporting Information, Supplementary data 1). Due to the large variability within the clinical groups (where some values can represent outliers) and the nonnormal data distribution of some variables, the median and median absolute deviation (Wilcox, 2017) were used to analyze demographic and cognitive characteristics (Table 1). Additionally, to solve the pronounced between-group variability (Supporting Information, Supplementary data 2), it was necessary to match the clinical groups in age, sex, and education using two subsamples of the HCs, both with *n* = 50 (Supporting Information, Supplementary data 3): the first subsample (HC_sub1_) was matched with bvFTD and svPPA patients, and the second subsample (HC_sub2_) was matched with CBS, nfvPPA, and PSP patients. Based on the clinical dementia rating (CDR), bvFTD and svPPA patients were in the mild stage of the disease (CDR = 1), while those with the other variants were in the early disease stage (CDR = 0.5) (see Table 1). However, all variants were comparable regarding their cognitive status, as assessed by the Mini-Mental State Examination (MMSE). Additionally, all variant groups were significantly different from the HC group on the CDR and MMSE scores (Table 1; see details in Supporting Information, Supplementary data 2). Among the variant groups, analysis of disease severity and cognitive measures presented statistically significant differences only between the bvFTD and nfvPPA groups in the CDR index.

**Table 1.** Demography, disease severity, and cognitive status

Group	*n*	Age	Sex (% of females)	Education	CDR	MMSE
HC_total_	99	66.0 (4.45)	54.5	18.0 (2.97) [4]	0 (0.0) [31]	30.0 (0)
HC_sub1_	50	63.0 (5.19)	46	16.0 (1.48) [1]	0 (0.0) [31]	29.0 (1.48)
HC_sub2_	50	68.0 (5.93)	58	17.0 (1.48) [2]	0 (0.0) [11]	29.5 (0.74)
bvFTD	47	62.0 (5.93)	38.3	15.5 (3.71)	1 (0.74)	25.0 (4.45)
CBS	38	67.0 (8.15)	52.6	16.0 (2.97) [2]	0.5 (0.37) [2]	25.0 (4.45) [4]
nfvPPA	34	69.5 (8.90)	52.9	16.0 (2.97)	0.5 (0.0) [1]	26.0 (2.97)
PSP	42	68.5 (6.67)	50	16.0 (2.97) [2]	0.5 (0.74) [3]	26.0 (2.97) [4]
svPPA	38	64.0 (7.41)	44.7	16.0 (2.97) [1]	1.0 (0.37)	24.5 (3.71) [1]
Shapiro–Wilk normality test		*p* = 0.264	—	*p* < 0.001	*p* < 0.001	*p* < 0.001
Significant between-group comparisons		a, b, c			a, d	d

*Note*. Descriptive statistics are presented as the median (median absolute deviation) [missing values]. The HC_sub1_ subsample is demographically matched with the bvFTD and svPPA groups, and the HC_sub2_ subsample is matched with the CBS, nfvPPA, and PSP variant groups. Group median comparisons were based on a 5,000 permutations test to deal with tied values (Wilcox, 2017); see details in Supporting Information Table S1. The *p* values were set at 0.05 and adjusted by the Bonferroni method. a: bvFTD vs. nfvPPA; b: bvFTD vs. PSP; c: PSP vs. svPPA; d: all variant groups presented statistically significant differences to the HC group. bvFTD = behavioral variant of FTD; CBS = corticobasal syndrome; CDR = Clinical Dementia Rating; HC = healthy controls; MMSE = Mini-Mental State Exam; nfvPPA = nonfluent variant primary progressive aphasia; PSP = progressive supranuclear palsy; svPPA = semantic variant primary progressive aphasia.

### MRI: Analysis

**Figure 1.**
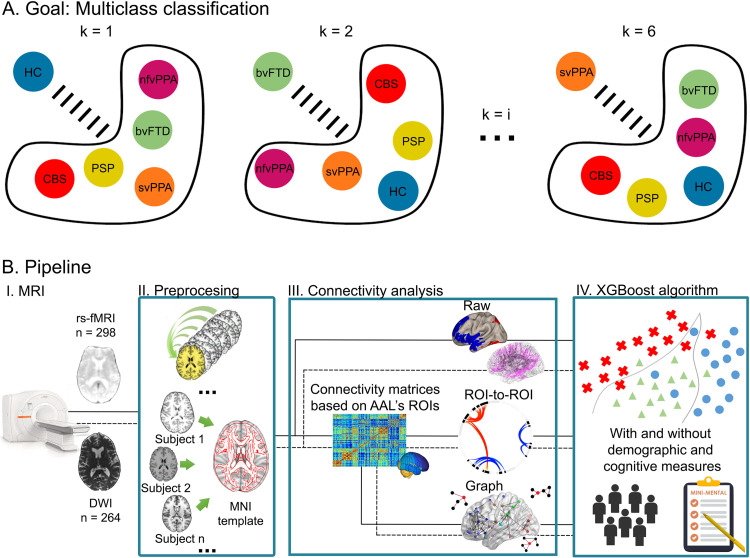
Goal and pipeline. (A) Graphic representation of multiclass classification where each group is differentiated from the other groups. In this case, there are six comparisons (k = 6). (B) Pipeline representation. I: MRI and the sequences employed. II: Preprocessing steps depended on the sequence type. III: Connectivity analysis included two modalities (functional and structural) and three methods (raw, ROI-to-ROI, and graph connectivity). IV: XGBoost algorithms were used for multiclass classification of groups, using both modalities and three methods of connectivity, with and without demographic and cognitive measures. bvFTD = behavioral variant of FTD; CBS = corticobasal syndrome; DWI = diffusion-weighted images; HC = healthy control; nfvPPA = nonfluent variant primary progressive aphasia; PSP = progressive supranuclear palsy; rs-fMRI; resting-state fMRI; svPPA = semantic variant primary progressive aphasia.

All MRI images were acquired from a 3.0 Tesla MR device following the protocols of the frontotemporal lobar degeneration neuroimaging initiative between 2008 and 2016. Although two different scanners were used (Siemens model Trio; scanner 1 = 285 subjects, 95.6 %; and scanner 2 = 13 subjects, 4.4 %), previous studies with this dataset have pointed out that this variability is negligible (Dickerson et al., 2008; Fox et al., 2012; Melzer et al., 2020; Noble et al., 2017a; Noble et al., 2017b; Zhou et al., 2018). In addition, only images with the same acquisition parameters for both scanners were selected to avoid additional sources of variability (Han et al., 2006; Mueller et al., 2011). Finally, additional procedures were performed to assess the potential effects of the scanner variability (see below). To enable a greater sample size, only cross-sectional data were included. We detail the study goal and the pipeline in Figure 1.

#### Resting-state fMRI (rs-fMRI): Connectivity matrices.

Three approaches were used to analyze the functional connectivity pattern in each variant group. First, connectivity was analyzed at the voxel level. We refer to this approach as raw connectivity, given that it is the most straightforward method to obtain functional connectivity maps (Nieto-Castanon, 2020). Second, at the ROI level, we analyzed the linear correlation between regions (ROI-to-ROI) because this is the gold standard for obtaining averaged voxel-wise associations (Fox & Raichle, 2007; Ibañez et al., 2021a, 2021b; Nieto-Castanon, 2020). Third, we employed graph connectivity measures to characterize brain network organization in a more comprehensive manner (Bressler & Menon, 2010; Rubinov & Sporns, 2010; Sporns, 2018). The three approaches were implemented to compare each variant with its respective HC subsample and between other variants based on *t* statistics. The multiple comparison problem was accounted for by using a threshold-free cluster enhancement (TFCE) method (Chen et al., 2018; Smith & Nichols, 2009), except for the graph measures, where we used the false discovery rate (FDR) method with *p* < 0.05.

The rs-fMRI signals were obtained from all subjects with an echo-planar pulse sequence and the following parameters: TR = 2,000 ms, TE = 27 ms, flip angle = 80 degrees, voxel size = 3 mm^3^, number of slices = 240. The preprocessing and functional connectivity analysis were implemented with the CONN 20.b toolbox (Whitfield-Gabrieli & Nieto-Castanon, 2012) running in SPM12 on MATLAB R2018b. For the functional images, the first five volumes were removed. The pipeline for preprocessing the images was set as the default of the CONN toolbox: (1) subject-motion estimation and correction, (2) automatic translations of the center to the (0, 0, 0) coordinates, (3) slice timing correction, (4) outlier detection (global signal *z*-value threshold = 5, subject-motion mm threshold = 0.9), (5) direct segmentation and normalization to Montreal Neurological Institute (MNI) space with a resolution of 3 mm^3^, (6) automatic translations of the center to the (0, 0, 0) coordinates of structural images, (7) direct segmentation and normalization to MNI of structural images (resolution of 1 mm^3^), (8) smoothing with a Gaussian kernel (8 mm × 8 mm × 8 mm), (9) denoising (linear regression of confounding effects of white matter, cerebrospinal fluid, realignment, and scrubbing), and (10) band-pass filter (0.001–0.09 Hz).

TFCE’s corrections were employed for raw and ROI-to-ROI connectivity because they can assess statistical significance at the voxel and ROI level without requiring one to set an arbitrary threshold, thus providing unbiased results (Chen et al., 2018; Smith & Nichols, 2009). Additionally, TFCE is more sensitive to both focal and peripheral effects than classical correction methods, reaching the best balance between family-wise error (FWE) rates and replicability (Chen et al., 2018). We calculated TFCE through 1,000 permutations and a significance level of *p* < 0.05 (FEW-corrected). Since the aggregation of areas within clusters (like in the case of the TFCE method) can interfere with the calculation of graph theory metrics (Bullmore & Sporns, 2009; Sporns, 2010), we employed the FDR correction which is the method of choice to correct for the multiple comparisons problem when using those measures (Agosta et al., 2014; Khazaee et al., 2015).

At the voxel level, the raw functional connectivity analysis included four measures to characterize the brain’s complexity (Mohanty et al., 2020). First, we employed global correlation, which represents the average of the correlation coefficient between each voxel and all other voxels in the brain (Nieto-Castanon, 2020). Second, we employed local correlation, which is defined as the average of the correlation coefficients of every voxel and their neighboring voxels in a kernel size of 30 mm (Deshpande et al., 2009). Additionally, two measures that are nonscale invariant were implemented to analyze BOLD signal power within a frequency window of interest (0.00–0.09 Hz). First, we used the amplitude of low-frequency fluctuations, considering the root-mean-square of the time series of each voxel after low- or band-pass filtering (Yang et al., 2007). Second, we used the fractional amplitude of low-frequency fluctuations to represent the power of the frequency band of interest (0.001–0.09 Hz) compared to the entire frequency spectrum. This measure represents the ratio of the root-mean-square of the BOLD signal at each voxel after vs. before the filtering (Zou et al., 2008).

At the ROI level, we extracted the mean time course of the BOLD signal of each one of the 116 regions according to the automated anatomical labeling (AAL) atlas (Tzourio-Mazoyer et al., 2002). An ROI-to-ROI connectivity matrix for each subject was calculated for all regions using the Fisher-transformed bivariate correlation coefficient between every pair of ROI time series (Nieto-Castanon, 2020). The TFCE clustering was based on a hierarchical algorithm method, where ROIs with similar effect patterns were grouped to achieve more meaningful results (Nieto-Castanon, 2020). Regarding graph measures, we included the positive values of the ROI-to-ROI connectivity matrices only, with a threshold of 0.3, to avoid small effect sizes (Cohen, 1988, 1992; Rosenthal, 1996). Thus, the AAL’s regions were considered the nodes, and the linear correlations greater than 0.3 the edges. The matrices obtained were used as undirected weighted inputs for the calculation of graph metrics in the brain connectivity toolbox (https://www.brain-connectivity-toolbox.net). We used the approach used in Sedeño et al. (2017) by employing weighted graph measures, given that the information about the connection strength is preserved (van Wijk et al., 2010). Following previous standards in this field (Rubinov & Sporns, 2010), seven graph connectivity metrics were analyzed for each ROI: (1) global efficiency: the average of inverse-shortest path (the minimum number of edges that must be traversed to go from one node to another) between this node and all other nodes in the network (Nieto-Castanon, 2020); (2) local efficiency: global efficiency computed on the neighborhood of the node (Nieto-Castanon, 2020); (3) degree: number of links connected to the node (Bullmore & Sporns, 2009); (4) strength: the sum of the weights of links connected to the node (Rubinov & Sporns, 2010); (5) clustering coefficient: fraction of node’s neighbors that are neighbors of each other (Bassett & Bullmore, 2006); (6) betweenness centrality: the fraction of all shortest paths in the network that contain a given node (Rubinov & Sporns, 2010).

#### Diffusion-weighted images (DWI).

Structural connectivity was analyzed in a similar way to functional connectivity, using the three previous methods to characterize connectivity changes at different levels. The DWI sequences were acquired for 264 of the subjects, including 77 HCs (HC_sub1_ = 43, HC_sub2_ = 41), 45 with bvFTD, 35 with CBS, 34 with nfvPPA, 37 with PSP, and 36 with svPPA. As in the total sample, no sociodemographic differences were observed (Supporting Information, Supplementary data 3). The group comparison methods for the three DWI approaches were the same as those used for the functional data. The multiple comparisons problem for the raw and graph measures of connectivity was corrected by the FDR method (*p* < 0.05). The connections between ROIs were corrected by the TFCE method using 1,000 permutations with *p* < 0.05 (FWE-corrected).

All DWI were acquired with an echo-planar sequence in two dimensions and 64 diffusion sampling directions, with the following parameters: TR = 7,400 ms, TE = 86 ms, flip angle = 180 degrees, in-plane resolution = 2.2 mm, slice thickness = 2.2 mm, and b-value = 2,000 s/mm^2^. The MRIToolkit toolbox (https://github.com/delucaal/MRIToolkit), running on Matlab R2018b was used for preprocessing, where the pipeline includes denoising based on principal components analysis (Veraart, Fieremans, & Novikov, 2016) and corrections for distortions due to head motion and eddy currents. The analysis was implemented with DSI Studio (version 2021, Jul 12, https://dsi-studio.labsolver.org). The quality of the DWIs was confirmed by an average of 0.89 (*SD* = 0.01) in the mean Pearson correlation coefficient of the “neighboring” DWI (Yeh et al., 2019b). To obtain the spin distribution function (Yeh et al., 2010), diffusion data were reconstructed in the MNI space using q-space diffeomorphic reconstruction (Yeh & Tseng, 2011) with a length ratio of 1.25 and a resolution of 2 mm isotropic. We used a deterministic fiber tracking algorithm (Yeh et al., 2013), which has been shown to accomplish 92% valid connections over an average of 54% of other algorithms (Maier-Hein et al., 2017).

The multiple comparison problem was tackled using a TFCE method (Chen et al., 2018; Smith & Nichols, 2009), except for the graph measures, in which we used the discovery rate (FDR) method with *p* < 0.05. The reasons for selecting these methods were the same as with functional connectivity, but for raw structural connectivity, we used the FDR correction. The FDR method is the gold standard for tackling the multiple comparisons problem in tractography (Whitwell et al., 2010a; Yeh et al., 2019b; Zhang et al., 2013) because it enables a substantial improvement of statistical power for comparing individuals averaging tracts (Schwartzman et al., 2008). Moreover, tract-crossing fibers (Tournier, 2010) can affect clustering measures, thus discouraging the use of TFCE corrections.

First, we initialized the analysis of raw structural connectivity taking into account all the tracts included in the structural connectome (Yeh et al., 2018). An automatic fiber tracking algorithm was used to calculate seven connectivity metrics for the subject’s tracts. The seeding region was placed at the track regions of the tractography atlas (Yeh et al., 2018) with a track-to-voxel ratio of 2. The anisotropy threshold, angular threshold, and step size were randomly selected, the last two between 15–90 degrees and 0.5–1.5 voxels, respectively (Yeh et al., 2018). Tracks with a length shorter than 30 or longer than 200 mm were discarded (Yeh et al., 2019a). Additionally, topology-informed pruning (Yeh et al., 2019a) was applied with 32 iterations to remove false connections. Seven raw connectivity measures were obtained for every tract using two models. On the one hand, we obtained four measures from the classical tensor model (Basser et al., 1994a, 1994b) with diffusion tractography image: (1) fractional anisotropy, degree of anisotropy of the diffusion process; (2) axial diffusivity: quantifies diffusivity along the principal axis of the tensor; (3) radial diffusivity: explains the diffusivity perpendicular to the principal axis of the tensor; and (4) mean diffusivity: characterizes the overall mean squared displacement of the water molecules (for more details on tensor-based measures calculations, see Hecke et al., 2016). On the other hand, from the q-space model (Callaghan, 1994) and the generalized q-sampling imaging method (Yeh et al., 2010, 2013), the following measures were obtained: (5) the normalized quantitative anisotropy: evaluates the most prominent fiber orientation, in a scaled way so that the maximum of each subject is one (Yeh et al., 2010, 2013); (6) the isotropic diffusion component derive: represents the non-directional restricted diffusion (Yeh et al., 2010, 2013); and (7) the restricted diffusion imaging: quantifies the density of restricted diffusion (Yeh et al., 2017).

Second, an ROI-to-ROI structural connectivity analysis was used to assess the integrity of tracts between gray matter regions. To this end, the fractional anisotropy associations among the 116 ROI of the AAL atlas were calculated (Tzourio-Mazoyer et al., 2002). A total of 10,000 seeds were placed in the whole brain (Yeh et al., 2010, 2013). The fractional anisotropy threshold was set at 0.2 for the elimination of voxels containing gray matter (Mori & van Zijl, 2002; Oishi et al., 2008). This value was chosen based on the existing literature on FTD structural connectivity characterization (Chen et al., 2020; Möller et al., 2015; Sheelakumari et al., 2020). Additionally, the angular threshold and step size were randomly selected from 15 to 90 degrees and 0.5 to 1.5 voxels, respectively (Yeh et al., 2018). The tracts that appeared repeatedly at a distance smaller than 1 mm, shorter than 30 mm, or longer than 200 mm were discarded (Yeh et al., 2019a, 2019b). Finally, one connectivity matrix was calculated for every subject based on the fractional anisotropy mean of the tracts that ended in every area of the 116 AAL’s ROIs. Furthermore, these matrices were used for the calculation of graph metrics, as explained for functional data.

### Machine Learning

We employed ML to compare classification performance of functional and structural connectivity data alone or in combination with demographic and cognitive measures. With this aim, we evaluated the individual contributions of the three methods of connectivity in the two modalities (functional and structural) to characterize the pathological groups. Each algorithm was executed two times, with connectivity data alone and with the demographic and cognitive features. Additionally, we created one model combining all methods and modalities. In total, 14 data-driven models were used for the classification of one class relative to the remaining subjects, following best practices in ML (Müller & Guido, 2016; Poldrack et al., 2019).

#### Feature engineering and selection.

The total number of multimodal features was on the order of 10^6^, voxel-wise variables entailing extensive computational time and memory requirements (Cohen et al., 2017; Huys et al., 2016). Using the full set of features might also induce adaptation of ML algorithms to the particularities of a specific dataset (overfitting), resulting in poor generalizability (Müller & Guido, 2016). Following previous procedures, we reduced dimensionality with group-level statistical analysis, also called filter method (Kassraian-Fard et al., 2016). Unlike other methods for dimensionality reduction, such as principal component analysis, this approach allows a more direct interpretation of the results (Huys et al., 2016; Pereira et al., 2009). In addition, filter methods are computationally inexpensive and do not take classifier performance into consideration (Kassraian-Fard et al., 2016). Moreover, our group-level statistical findings can be compared with previous research (Agosta et al., 2012; Bharti et al., 2017; Iaccarino et al., 2015; Popal et al., 2020; Tovar-Moll et al., 2014; Upadhyay et al., 2016; Whitwell et al., 2009). This hybrid approach has been employed in previous studies (Dottori et al., 2017; Fittipaldi et al., 2020; Gonzalez Campo et al., 2020; Kassraian-Fard et al., 2016; McMillan et al., 2012).

Each possible group comparison was calculated for each modality and method, with a statistical power threshold of 0.80 for detecting medium effect sizes, as recommended (Cohen, 1988); see Supporting Information, Supplementary data 2 for details. The significant results from these comparisons were used as inputs for the ML algorithms in our training sample. In the case of raw functional connectivity, the results were averaged for significant clusters (with a size greater than 50 voxels) across the individual maps of connectivity. To extract the most relevant features, we performed a progressive feature elimination approach in the training set (80% of the total sample, further details below) to select the optimum set of features after stabilization (Donnelly-Kehoe et al., 2018) using a k-fold scheme (with *k* = 5) with nested training and validation. At each iteration, the Gini scores were used to eliminate the features of the lowest importance, while evaluating feature stability on each nested fold. The variability of the feature ranking in the importance list was evaluated across nested k-folds. To this end, we assessed if the confidence interval’s right tail of each feature was ranked in the same way as the feature mean (see Supporting Information, Supplementary data 4 for analysis output details). Finally, we kept the N first features in the ranking, where N is the optimal number of features such that using more than N features fails to improve classifier performance.

#### Classification models.

Based on the selected features, we used the XGBoost algorithm (Kaufmann et al., 2019) to classify the different clinical groups. The XGBoost algorithm is a gradient boosting machine implementation that provides parallel computation tree boosting, enabling fast and accurate predictions and advanced regularization techniques to avoid overfitting (Torlay et al., 2017). This algorithm has proven successful in several diagnostic applications (Behravan et al., 2018; Torlay et al., 2017; Zheng et al., 2017). Gradient boosting machine is based on the gradient boosting technique, in which ensembles of decision trees iteratively attempt to correct the classification errors of their predecessors by minimizing a loss function (i.e., a function representing the difference between the estimated and true values) pointing in the negative gradient direction (Mason et al., 1999). When compared to other GBM algorithms, XGBoost provides regularized boosting, helping to reduce overfitting with more generalizable results (Torlay et al., 2017; Xuan et al., 2019).

Following best practices in ML (Müller & Guido, 2016; Poldrack et al., 2019), we used 80% of the sample for training and validation and 20% of the sample for testing. Within the training set, we performed k-fold cross-validation (with k = 5 nonoverlapping folds) in alternating nested training sets and validation sets to tune hyperparameters. The 20% left was an independent test set to perform an unbiased and accurate performance estimation. XGBoost has several hyperparameters, including the number of subtrees to retain, maximum tree depth, learning rate, minimum loss reduction required to further partition a leaf node, maximum number of leaves, and regularization weights (Wade, 2020). To choose the best hyperparameter combination, we used Bayesian optimization, an approach with demonstrated applicability to different problem settings (Feurer & Hutter, 2019; Zeng & Luo, 2017). This is an iterative algorithm with two key components: a probabilistic surrogate model and an acquisition function to decide which point to evaluate next. At each step, a new point in the hyperparameter space to explore is selected to be the maximum of an activation function of the prior knowledge and the uncertainty. As this optimization progresses, the chances of finding a better solution increase. Compared to other techniques, such as grid search (which is undermined by issues of dimensionality) or random search (where each guess is independent of the previous run), the Bayesian optimization algorithm is fast to compute, enabling a thorough optimization of the hyperparameters.

The performance of the classifiers was evaluated through receiver operating characteristic (ROC) curves (Fawcett, 2006), in which the sensitivity (true positive rate) and (1 − specificity) (i.e., false-positive rate) were used as the Y and X axes, respectively. These results were condensed using the area under the ROC curve (AUC) value, representing the probability that a randomly picked subject from the correct group will have a higher score according to the classifier than a randomly picked subject from the incorrect group (Müller & Guido, 2016). The pipeline divided the original dataset into six binary datasets, where the positive class in each of these datasets corresponded to the group of interest, and the negative class was composed of all the other groups. Then, for each dataset, performance was evaluated through the microaverage AUC by averaging classification performance with respect to the labels and samples, thus taking into account the imbalance between classes and producing an unbiased performance metric (Koyejo et al., 2015).

## RESULTS

### Functional Connectivity

**Figure 2.**
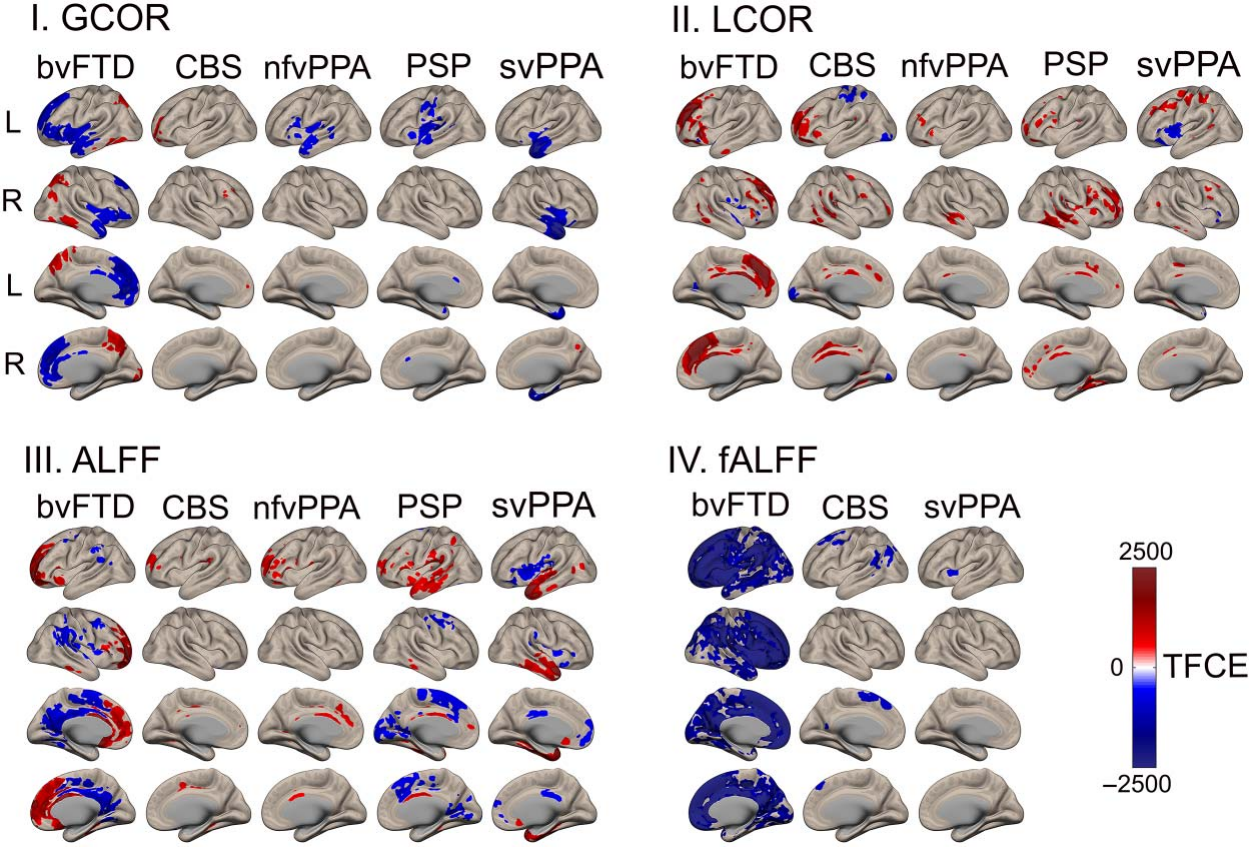
Raw functional connectivity in five frontotemporal dementia variants. Each variant group was compared to a subsample of the healthy control group matched for sex, age, and years of education. Connectivity metrics were calculated at the voxel level and corrected by TFCE, *p*_FEW_ < 0.05, where clusters represent statistically significant differences with respect to the healthy controls. ALFF = amplitude of low-frequency fluctuations; bvFTD = behavioral variant; CBS = corticobasal syndrome; fALFF = fractional amplitude of low-frequency fluctuations; L = left; nfvPPA = progressive nonfluent aphasia; PSP = progressive supranuclear palsy; R = right; svPPA = semantic variant of FTD; TFCE = threshold-free cluster enhancement.

**Figure 3.**
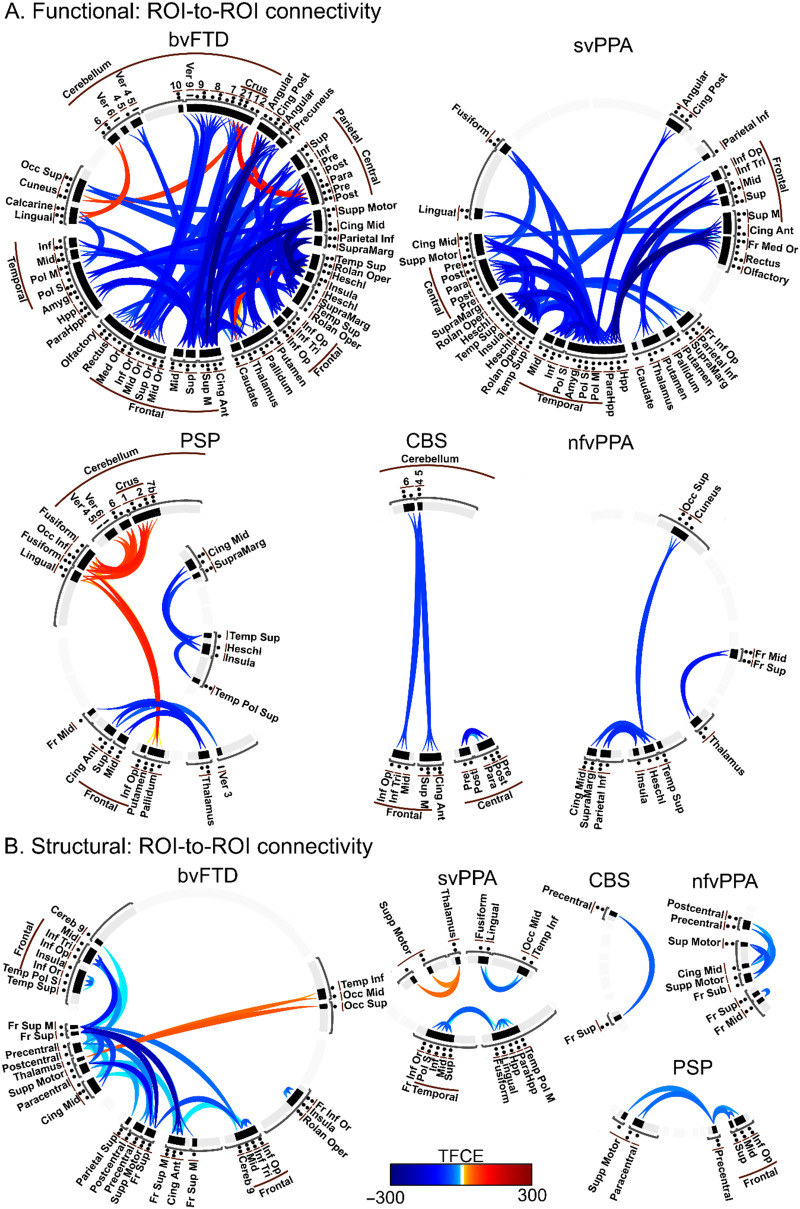
ROI-to-ROI connectivity in five variants of frontotemporal dementia. Functional connectivity (A) employing linear correlation among ROIs, while structural connectivity (B) using fractional anisotropy means of the tracts that end in each ROI. Each variant group was compared with a subsample of the HC group matched for sex, age, and years of education based on the 116 ROIs in the AAL atlas. Multiple comparisons were corrected by TFCE, *p*_FEW_ < 0.05, where ROIs with similar effect patterns were grouped through a hierarchical algorithm method to achieve more meaningful results. Ant = anterior; Amyg = amygdala; bvFTD = behavioral variant; CBS = corticobasal syndrome; Cereb = cerebellum; Cing = Cingulum; Fr = frontal; Hpp = hippocampus; Inf = inferior; L = left; M = medial; Mid = middle; nfvPPA = progressive nonfluent aphasia; Occ = occipital; Op = opercular; Oper = operculum; Or = orbital; Pol M = middle pole; Pol S = superior pole; Post = posterior; PSP = progressive supranuclear palsy; R = right; Sup = superior; Supramarg = supramarginal; Supp = supplementary; svPPA = semantic variant of FTD; TFCE = threshold-free cluster enhancement; Temp = temporal; Tri = triangularis; Ver = vermis.

**Figure 4.**
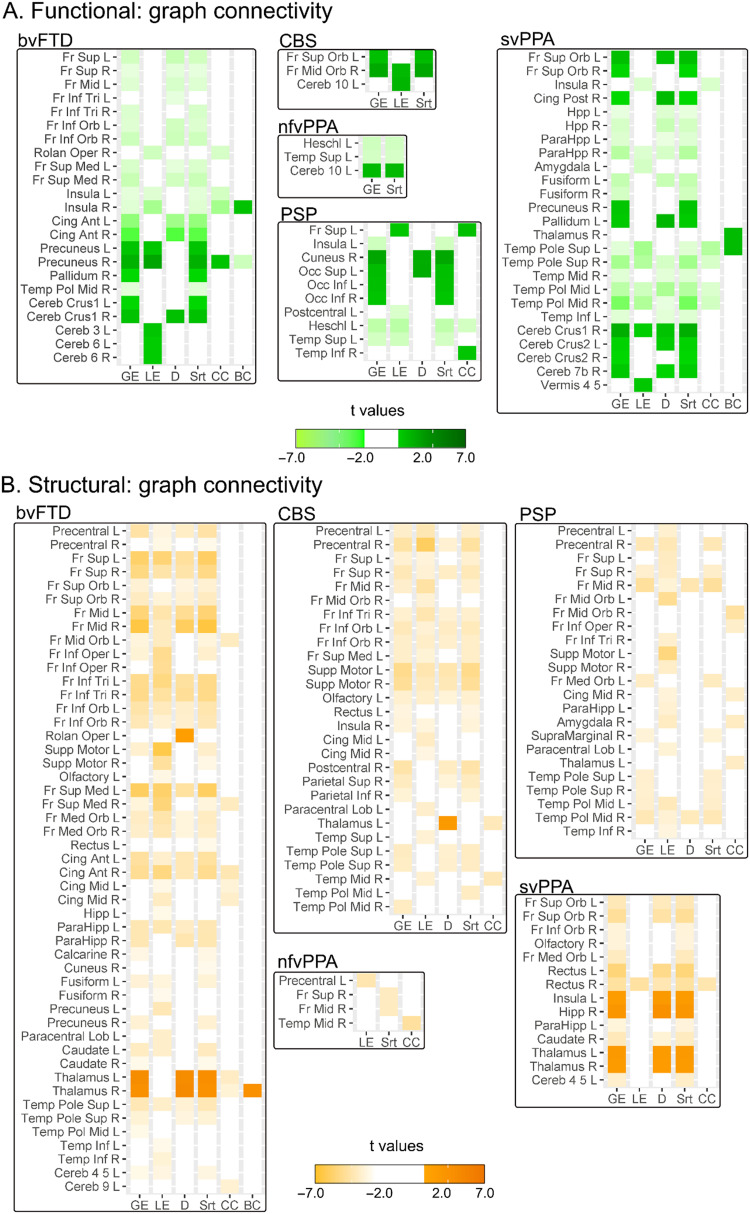
Graph connectivity in five variants of frontotemporal dementia. The 116 ROIs in the AAL atlas were considered the nodes. The edges of functional connectivity (A) were linear correlation coefficients greater than 0.3, and those of structural connectivity (B) were the fractional anisotropy means of the tracts that ended in every ROI with a threshold of 0.2. Each variant group was compared with a subsample of the healthy control group matched for sex, age, and years of education based on the 116 ROIs in the AAL atlas. Multiple comparisons were corrected by FDR. Ant = anterior; BC = betweenness centrality; bvFTD = behavioral variant; CBS = corticobasal syndrome; CC = clustering coefficient; Cereb = cerebellum; Cing = cingulum; D = degree; Fr = frontal; GL = global efficiency; Hpp = hippocampus; Inf = inferior; L = left; LE = local efficiency; Med = medial; Mid = middle; nfvPPA = progressive nonfluent aphasia; Op = opercular; Oper = operculum; Orb = orbital; PSP = progressive supranuclear palsy; R = right; svPPA = semantic variant of FTD; Str = strength; Sup = superior; Supp = supplementary; Temp = temporal; Tri = triangularis.

Depending on the method used, each variant group exhibited heterogeneous functional network alterations. The patients with bvFTD presented multiple changes in their frontal networks, as detected by raw (Figure 2), ROI-to-ROI (Figure 3A), and graph (Figure 4A) connectivity measures. Additionally, clusters in the insula and anterior temporal area were significantly disconnected from distant areas but not from nearby voxels (Figure 2). Additionally, temporal regions (ROI-to-ROI) were less connected (Figure 3A), and cerebellar areas presented improved network organization (Figure 4A). Raw functional connectivity in the CBS group showed an increase with the left prefrontal areas (Figure 2). In contrast, the ROI-to-ROI method detected impaired connectivity of the cerebellum with the frontal regions (Figure 3A), while efficiency and strength graph properties of the orbitofrontal areas and cerebellum increased (Figure 4A). In the nfvPPA group, there was evidence of a decrease in the global correlation between the insula and anterior temporal region in the left hemisphere (Figure 2). Based on the ROI-level analysis, the same areas showed decreased connectivity with occipital and parietal regions (Figure 3A). The graph connectivity analysis showed a diminution of the global efficiency and strength values in the left Heschl gyrus and left superior temporal regions (ROI), with the same metrics increasing in the cerebellum (Figure 4A). Regarding the PSP group, raw functional connectivity showed alterations in the left insula, cerebellum, and inferior and superior temporal gyri (Figure 2 and Supporting Information, Supplementary data 5). ROI-to-ROI connectivity analysis showed increased cerebellar connections with the occipital lobe (Figure 3A). Graph analyses demonstrated better network organization in the bilateral occipital ROIs and a deterioration of network organization in the insula, Heschl’s area, and temporal superior area in the left hemisphere (Figure 4A). Regarding the svPPA patients, raw functional connectivity analyses evidenced impaired connectivity of the bilateral anterior temporal gyri and the insula (Figure 2). The ROI-to-ROI connectivity showed decreases in multiple connections (intratemporal, tempo-occipital, tempo-central, and fronto-subcortical connections; see Figure 3A). Moreover, the graph connectivity analysis showed increases mainly in global efficiency, degree, and strength metrics in the bilateral frontal, superior, orbital, and cerebellar ROIs (Figure 4A).

### Structural Connectivity

**Figure 5.**
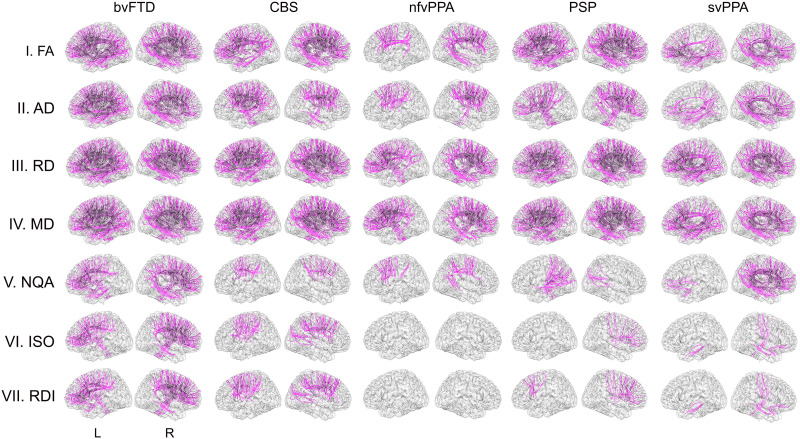
Raw structural connectivity in five frontotemporal dementia variants. Each variant group was compared to a subsample of the healthy control group matched for sex, age, and years of education. Connectivity was analyzed with a determinist tractography method, and multiple comparisons were controlled for by FDR (*p*_FDR_ < 0.05). The results showed tracts significantly different from healthy controls. AD = axial diffusivity; bvFTD = behavioral variant; CBS = corticobasal syndrome; FA = fractional anisotropy; ISO = isotropic diffusion component derived; L = left; MD = mean diffusivity; NQA = normalized quantitative anisotropy; nfvPPA = progressive nonfluent aphasia; PSP = progressive supranuclear palsy; R = right; RD = radial diffusivity; RDI = restricted diffusion imaging; svPPA = semantic variant of FTD.

Similar to functional connectivity, structural networks evidenced variable results depending on the method employed. The patients with bvFTD showed several altered tracts based on the raw structural connectivity analysis (Figure 5). Similar findings were obtained in the ROI-to-ROI connectivity analysis (Figure 3B), indicating impairments primarily in the fronto-frontal connections but also in the fronto-cerebellar and fronto-central connections. Similarly, all frontal ROIs presented decreased network graph organization (Figure 4B). The CBS group had a similar pattern to the bvFTD group in raw structural connectivity (Figure 5) but with more extended changes across the cerebellar connections (Supporting Information, Supplementary data 5). The ROI-to-ROI connectivity analysis showed decreases in the precentral and frontal superior regions in the right hemisphere (Figure 3B). The analysis of the graph metrics presented diminished efficiency, degree, and strength metrics in the frontal and temporal regions (Figure 4B). In the nfvPPA group, reduced raw structural connectivity was observed in the corpus callosum, frontal aslant tract, superior longitudinal fasciculus, anterior thalamic radiations, superior thalamic radiations, and cerebellar connections (Figure 5 and Supporting Information, Supplementary data 5). ROI-to-ROI connectivity presented decreases between the left frontal and right central areas and between the superior and middle right frontal ROIs (Figure 3B). These ROIs showed reduced strength, while local efficiency and the clustering coefficient decreased in the left precentral and right mid temporal ROIs, respectively (Figure 4B). The patients with PSP presented a similar pattern to those with bvFTD in raw structural connectivity (Figure 5), with numerous additional alterations in the cerebellar connections (Supporting Information, Supplementary data 5). Additionally, this group presented disconnections between the right precentral ROI to frontal regions (Figure 3B). The network organization in the frontal, central, and temporal ROIs was reduced, mainly regarding efficiency and strength (Figure 4B). In the svPPA group, the three methods mainly evidenced a loss of connectivity of the temporal lobes with the rest of the brain. All raw connectivity indices showed significant differences in the uncinated fasciculus and cingulum parahippocampal tract (Figure 5 and Supporting Information, Supplementary data 5). ROI-to-ROI connectivity analyses (Figure 3B) detected a decrease in connectivity of the intrafrontal and intratemporal areas in the right hemisphere (Figure 3B). Global efficiency, degree, and strength values increased in the bilateral thalamic, left insular, and right hippocampal ROIs but decreased in the frontal and cerebellar regions (Figure 4B).

### Machine Learning

#### Multiclass classification.

**Figure 6.**
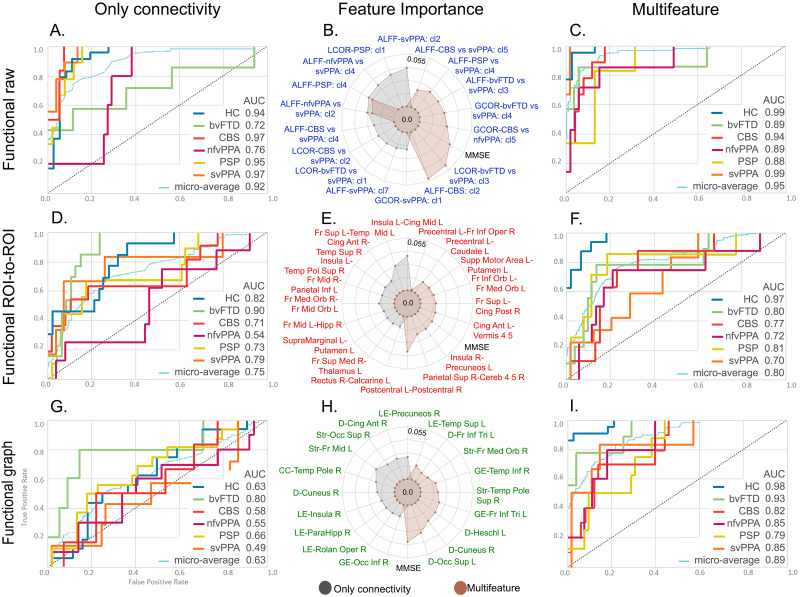
XGBoost classifier performance with functional connectivity data for the multiclass classifications of five variants of FTD and HCs. The rows correspond to the three methods used for the analyses of functional connectivity: raw, ROI-to-ROI, and graph connectivity. The first column corresponds to the performance of models with only connectivity data from the three methods (A, D, and G). The second column presents the feature importance for both models corresponding to the same method (B, E, and H). The third column shows the multifeatured approach where connectivity data were used along with demographic and cognitive variables (C, F, and I). Additional details on classification performance are provided in Supporting Information, Supplementary data 4. ALFF: amplitude of low-frequency fluctuations; Ant = anterior; AUC = area under the ROC curve; bvFTD = behavioral variant; CBS = corticobasal syndrome; CC = clustering coefficient; Cereb = cerebellum; Cing = cingulum; cl = cluster; D = degree; Fr = frontal; GCOR = global correlation; GL = global efficiency; HC = healthy controls; Hipp; hippocampus; Inf = inferior; L = left; LCOR = local correlation; LE = local efficiency; nfvPPA = progressive nonfluent aphasia; Med = medial; Mid = middle; MMSE = Mini-Mental State Examination; Occ = occipital; Oper = operculum; Orb = orbital; PSP = progressive supranuclear palsy; R = right; Rolan = rolandic; Sup = superior; svPPA = semantic variant of FTD; Str = strength; Temp = temporal.

The multiclass classification among groups based on connectivity data from the raw functional method achieved an AUC of 0.92 (Figure 6A). The model with multimodal data, including raw functional connectivity and cognitive features, reached the highest classification performance (AUC = 0.95) (Figure 6C). The top-ranked features in both models included the amplitude of low-frequency fluctuations and global and local correlation metrics. Furthermore, in the multimodal approach, the MMSE score was the fourth ranked in the feature importance list (Figure 6B). The models with ROI-to-ROI functional connectivity features yielded AUCs of 0.75 and 0.80 when the connectivity data were analyzed individually (Figure 6D) or with the multimodal approach (Figure 6F), respectively. The top features in these models are shown in Figure 6E, in which the MMSE score ranked third in the multimodal model. The model with functional graph connectivity data only yielded an AUC of 0.63 (Figure 6G), a value that was increased in its multimodal counterpart to 0.89 (Figure 6I). Finally, Figure 6H shows the selected features for the graph models and their relative feature importance, where the MMSE score was the most important feature in this multimodal approach.

**Figure 7.**
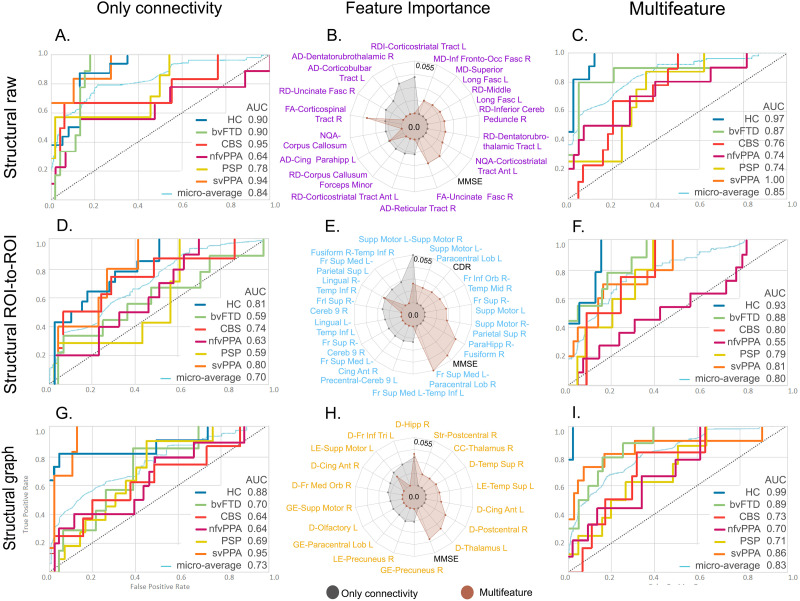
XGBoost classifier performance with structural connectivity data for the multiclass classifications of five variants of FTD and HCs. The rows correspond to the three methods used for the analyses of functional connectivity: raw, ROI-to-ROI, and graph connectivity. The first column corresponds to the performance of models with connectivity data only from the three methods (A, D, and G). The second column presents the feature importance for both models corresponding to the same method (B, E, and H). The third column shows the multifeatured approach where connectivity data were used along with demographic and cognitive variables (C, F, and I). Additional details on classification performance are provided in Supporting Information, Supplementary data 4. Ant = anterior; AUC = area under the ROC curve; bvFTD = behavioral variant; CBS = corticobasal syndrome; CC = clustering coefficient; Cereb = cerebellum; Cing = cingulum; D = degree; FA = fractional anisotropy; Fasc = fasciculus; Fr = frontal; GL = global efficiency; HC = healthy controls; Hipp = hippocampus; Inf = inferior; L = left; LE = local efficiency; Long = longitudinal; MD = mean diffusivity; Med = medial; NQA = normalized quantitative anisotropy; nfvPPA = progressive nonfluent aphasia; MMSE = Mini-Mental State Examination; Orb = orbital; PSP = progressive supranuclear palsy; R = right; RD = radial diffusivity; Sup = superior; Supp = supplementary; svPPA = semantic variant of FTD; Str = strength; Temp = temporal; Tri = pars triangularis.

The model with raw structural data achieved an AUC of 0.84 (Figure 7A), while its multimodal counterpart reached an AUC of 0.85 (Figure 7C). The top features included in these models and their importance are shown in Figure 7B. The MMSE score feature ranked as one of the top features in the multimodal model. In the case of the model with ROI-to-ROI structural connectivity only, an AUC performance of 0.70 was obtained (Figure 7D), a value that increased to 0.80 when incorporating multimodal features (Figure 7F). The feature importance is shown in Figure 7E, where the MMSE score was the second most important feature in the multimodal version. The models with graph connectivity data presented AUC values of 0.73 and 0.83 using connectivity data only (Figure 7G) and when combined with multimodal data (Figure 7I), respectively. The top features of these models are presented in Figure 7H, where the MMSE score was the most relevant feature in this multimodal approach.

**Figure 8.**
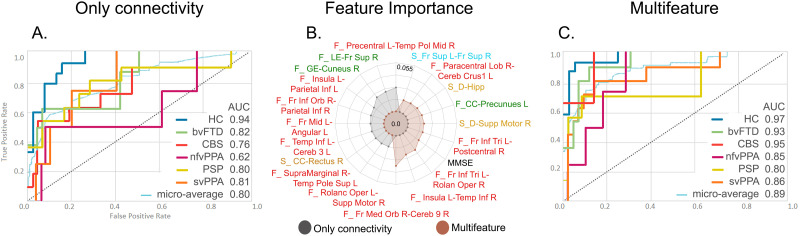
XGBoost classifier performance combining functional and structural connectivity for the multiclass classification of five variants of FTD and HCs. Performance with multimodal (A) and multifetured data (C) is presented individually, as well as the feature importance for both models (B). Additional details on classification performance are provided in Supporting Information, Supplementary data 4. AUC = area under the ROC curve; bvFTD = behavioral variant; CBS = corticobasal syndrome; CC = clustering coefficient; Cereb = cerebellum; D = degree; F = functional; Fr = frontal; GL = global efficiency; HC = healthy controls; Hpp = hippocampus; Inf = inferior; L = left; LE = local efficiency; nfvPPA = progressive nonfluent aphasia; Med = medial; Mid = middle; MMSE = Mini-Mental State Examination; Oper = operculum; Orb = orbital; PSP = progressive supranuclear palsy; R = right; Rolan = rolandic; S = structural; Sup = superior; sup = supplementary; svPPA = semantic variant of FTD; Temp = temporal; Tri = pars triangularis.

Finally, the combination of all connectivity data from both modalities and the three methods for multiclass classification reached an AUC of 0.80 (Figure 8A). When incorporating cognitive features, we obtained an AUC of 0.89 (Figure 8C). The top features are shown in Figure 8B. Of note, the MMSE score ranked fourth in the multimodal approach. Results for the average AUC for each class in the ROC curves showed varying mean performance across modalities and methods (Figures 6, 7, and 8). Nevertheless, performance variability was low on different folds as shown in the confidence intervals. Moreover, feature stability was assessed on nested k-folds during the validation step (see Supporting Information, Supplementary data 4 for details).

#### Comparison of metrics across modalities, methods, and techniques.

**Figure 9.**
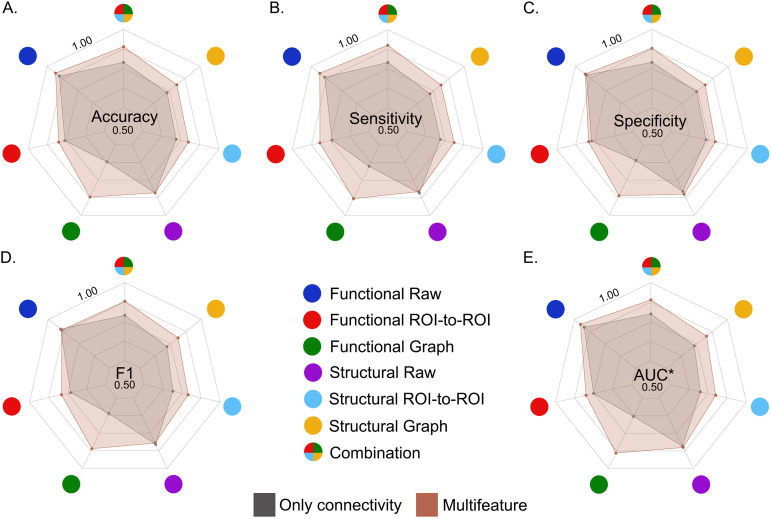
Performance metrics of the ML models. Mean for accuracy (A), sensitivity (B), specificity (C), F1 (D), and AUC (E) for the 14 ML models employed. *Microaverage AUC.

In total, 14 XGBoost data-driven models were used for the multiclass classification of the five variants of FTD and HCs based on the optimal feature sets after recursive optimization. The data were computed individually and combined with all modalities and methods (2 modalities × 3 methods + 1 multimodal). Furthermore, each of the models was calculated twice, with and without demographic and cognitive variables. Based on the average performance indicators (i.e., accuracy, sensitivity, specificity, F1, and AUC; Figure 9 and Supporting Information, Supplementary data 4), the top three performing models were the raw functional multifeatured model, followed by the raw functional connectivity model and the multimodal multifeatured model. To statistically compare the performance results, we employed nonparametric tests to assess statistically significant differences between the ROC curves (Venkatraman, 2000). In this approach, the equality of the curves is analyzed at all operating points, and a reference distribution is generated by permuting the pooled ranks of the test scores for each classification. We found that although the top performing model was the raw functional multifeatured model, the difference between this model and the two that followed (raw functional connectivity and multimodal multifeatured models) was not statistically significant (*p* > 0.05 in both cases).

**Figure 10.**
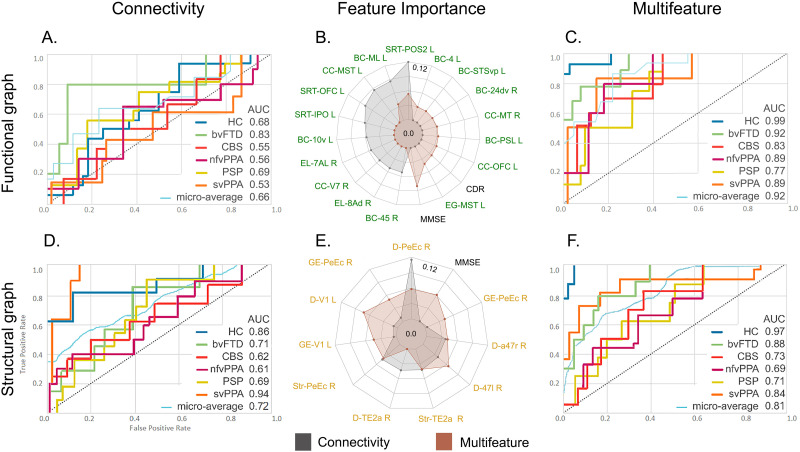
XGBoost classifier performance with graph connectivity data for the multiclass classifications of five variants of FTD and HCs using Human Connectome Project atlas parcellation. The rows correspond to the two modalities: functional and structural. The first column corresponds to the performance of models with connectivity data only (A and D). The second column presents the feature importance (B and E). The third column shows the multifeatured approach where connectivity data were used along with demographic and cognitive variables (C and F). 10v = area 10v (anterior cingulate and medial prefrontal cortices); 24dv = ventral area 24d (paracentral and mid cingulate cortices); 4 = primary motor cortex; 45 = Area 45 (inferior frontal cortex); 47l = area 47 lateral (inferior frontal cortex); 7AL = lateral area 7A (superior parietal cortex); 8Ad (dorsolateral prefrontal cortex); AUC = area under the ROC curve; a47r = area anterior 47r (inferior frontal cortex), bvFTD = behavioral variant; CBS = corticobasal syndrome; D = degree; GL = global efficiency; HC = healthy controls; IP0 = area intraparietal 0; L = left; MMSE = Mini-Mental State Examination; MST = medial superior temporal area; MT = middle temporal area; nfvPPA = progressive nonfluent aphasia; OFC = orbital frontal complex; PeEc = perirhinal ectorhinal cortex; POS2 = parieto-occipital sulcus area 2; PSL = perisylvian language area; PSP = progressive supranuclear palsy; R = right; STSvp = superior temporal sulcus ventral posterior; svPPA = semantic variant of FTD; TE2a = temporal area 2 anterior; V1 = primary visual cortex; V7 = seventh visual area.

To discard any possible effect due to the scanner, the classifiers were trained again with data acquired on only one scanner (Supporting Information, Supplementary data 4) with the 95.6 % of the data. The performance of all classifiers for one or two scanners did not show statistical differences in the AUC values (all *p* > 0.05). To check possible biases due to specific brain parcellations, we compared the performance of four of our models (graph connectivity and graph multifeatured in both modalities) using the Human Connectome Project (HCP) atlas (Glasser et al., 2016) with respect to our results with AAL atlas. Figure 10 shows the performance of ML models based on HCP parcellation with connectivity data only (Figure 10A and D) and multifeatured data (Figure 10C and F). Also, the top features are presented in Figure 10B and E. No significant differences were observed in the microaverage AUC for the functional graph connectivity (statistic = 1.62, *p* = 0.14), the functional graph multifeature (statistic = 2.12, *p* = 0.11), the structural graph connectivity (statistic = 1.58, *p* = 0.17) or the structural graph multifeatured (statistic = 1.75, *p* = 0.12) classifiers. These comparisons were based on nonparametric tests to assess statistical differences between the ROC curves (Venkatraman, 2000). Moreover, the most important features were in line with the results obtained with the AAL atlas. Areas from the prefrontal cortex (inferior, dorsolateral, and anterior cingulate regions), temporal lobe (superior and middle regions), and occipital lobe (superior and inferior regions) were evidenced in both parcellations of the functional connectivity classifiers. Similarly, both atlases’ structural classifiers shared the main features, including areas of frontal (inferior region) and occipital lobes.

## DISCUSSION

In this study, a simultaneous multiclass categorization of each FTD variant and healthy controls achieved a performance up to an AUC of 0.95. This was accomplished with a multifeatured strategy, where the classifiers combining brain network connectivity and cognitive assessments increased model performance. The multimodal classifiers evidenced the relative importance of specific domains for FTD variant characterization. Through progressive feature elimination, an optimum set of features was obtained by removing redundant and uninformative variables. The results address current calls for robust FTD variant multimodal marker classification. This approach, if further replicated and validated, may be translated into the development of future affordable clinical decision computational tools.

Our framework provided support for proposed hypotheses regarding the multiclass classification of FTD variants based on computational inference. First, we obtained highly accurate simultaneous multiclass classification of each FTD variant relative to other variants and controls after feature optimization. In line with previous studies, the functional ROI-to-ROI models showed alterations in fronto-temporal (Jastorff et al., 2016; Meijboom et al., 2017), intrafrontal (Dopper et al., 2014; Whitwell, 2019), and precuneus-insula (Whitwell et al., 2011b) connectivity. Moreover, the functional graph theory models captured node-degree differences in the left superior occipital area (Agosta et al., 2013; Reyes et al., 2018), left Heschl gyrus (Agosta et al., 2013), and left frontal inferior pars triangularis (Zhou et al., 2012). Regarding the raw structural connectivity models, and in agreement with previous research, we found alterations in the uncinate fasciculus (Agosta et al., 2012; Daianu et al., 2016; Iaccarino et al., 2015; Nguyen et al., 2013), superior longitudinal fasciculus (Agosta et al., 2012; Daianu et al., 2016), corpus callosum (Tovar-Moll et al., 2014), dentatorubrothalamic tract (Whitwell et al., 2011a), and inferior fronto-occipital fasciculus (Meijboom et al., 2017). Second, adding cognitive features (i.e., multifeatured approach) increased the averaged AUC performance metrics across all subject groups. The relevance of adding cognitive features was further evidenced in the feature importance lists of our models, where they ranked in the top four positions across the multifeatured models. Third, although the raw functional models ranked higher than the multimodal approach, the differences in performance were not statistically significant. This may be because the top features of the multimodal approaches were mainly functional connectivity features and adding information from other domains was not relevant for FTD variant characterization. Increased model complexity with a limited dataset may induce overfitting (Müller & Guido, 2016). Therefore, model performance may be lower. However, the multimodal approach helps to provide information on specific functional and structural alterations capturing differential patterns in FTD (Agosta et al., 2012; Nguyen et al., 2013; Reyes et al., 2018; Whitwell, 2019).

The feature importance lists from our ML analyses showed that the most relevant features for discriminating between FTD variants were generally in line with previous research. The subjects with bvFTD showed reduced connectivity in the prefrontal, insular, and temporal regions in terms of the functional (Agosta et al., 2013; Jalilianhasanpour et al., 2019; Seeley et al., 2009; Whitwell, 2019) and structural (Dopper et al., 2014; Mahoney et al., 2015; Tovar-Moll et al., 2014; Whitwell, 2019) networks, as well as a reduction in global network degree and efficiency (Filippi et al., 2017; Reyes et al., 2018; Saba et al., 2019; Sedeño et al., 2016). The primary alterations in the subjects with nfvPPA were observed in speech-language regions (Mandelli et al., 2016, 2018), with a predominance in the left hemisphere (Whitwell, 2019). The subjects with svPPA exhibited distinct patterns of disconnection in functional (Agosta et al., 2014; Popal et al., 2020; Whitwell, 2019) and structural (Agosta et al., 2012; Iaccarino et al., 2015; Zhang et al., 2013) connectivity in the temporal lobe. Last, for the subjects with CBS, we found alterations in motor/parietal areas (Tovar-Moll et al., 2014; Whitwell et al., 2014), while the subjects with PSP showed structural alterations in connections encompassing the thalamus (Borroni et al., 2014; Whitwell et al., 2011a). We also detected increased connectivity values in specific FTD variants, for example, in the connections involving the parietal lobe in bvFTD (Meijboom et al., 2017; Whitwell et al., 2011b), the frontal lobe in CBS (Wolpe et al., 2014), occipital lobe in PSP (Whitwell et al., 2011a), and structural thalamic tract connectivity in bvFTD and svPPA. These findings may reflect compensatory mechanisms as a result of the disconnection of critical brain regions specific to each pathology (Jalilianhasanpour et al., 2019; Saba et al., 2019). Overall, our ML approach was consistent with previous studies, while allowing the detection of specific alterations in distinct FTD variants with overlapping pathophysiological profiles, avoiding possible methodological biases. Furthermore, we compared, for the first time, the performance reached for multiclass classifications of FTD variants with data from different modalities of connectivity and using different methods.

Our approach provides a comprehensive computational framework that may be used in clinical settings after replication and external validation. Historically, ML research on the categorization of dementia has relied on binary comparisons and atrophy metrics. However, atrophy is associated with late-stage neurodegeneration (Lu et al., 2013; Seeley et al., 2008), while brain connectivity alterations may be present at early stages (Dopper et al., 2014; Meeter et al., 2017). The few studies with multiclass comparisons of FTD variants were conducted only with atrophy metrics (Kim et al., 2019) and tractography (Torso et al., 2020, 2021). Indeed, the literature examining binary comparisons of FTD variants is more extensive, individually assessing atrophy (Bachli et al., 2020; Bisenius et al., 2017; Meyer et al., 2017; Salvatore et al., 2014; Santillo et al., 2013) and functional (Moguilner et al., 2021) and structural (Mahoney et al., 2014; Santillo et al., 2013) connectivity measures. To the best of our knowledge, our approach is the first to enable multiclass model characterizations in a multimodal context. Moreover, this approach outperforms previous attempts for multiclass classification (Kim et al., 2019; Torso et al., 2020, 2021). Thus, our research lays the groundwork for the future creation of a useful clinical computational inference tool.

### Limitations and Future Studies

Our work has some limitations. First, despite the larger sample size compared to similar previous studies in the literature (Kim et al., 2019; Torso et al., 2020), the sample was based on a unique database. Future research may include multicentric samples from different consortia, with a variety of MRI acquisition protocols to assess the robustness of this method against heterogeneity. Additionally, to test the robustness of our results against sample heterogeneity, data from underrepresented populations with different genetic, demographic, and socioeconomic factors should be included (Ibañez et al., 2021a, 2021b). Second, we lacked histopathological diagnosis confirmation. However, this limitation is shared with previous similar work in the literature (Ma et al., 2020; Manera et al., 2019; Torso et al., 2020; Yu & Lee, 2019), and our approach can be extended to other datasets with histopathological confirmation. Third, our models should be compared in future studies to standard biomarkers such as PET and plasma indicators to evaluate potential synergistic biomarker combinations. Fourth, although the cognitive test employed in this study was the MMSE, additional specific assessments for FTD are available, such as particular executive function and language tasks (Custodio et al., 2016; Kramer, Alioto, & Kramer, 2020; Torralva et al., 2009; Younes & Miller, 2020), which may be added to the model. Fifth, despite the AAL being one of the most widely assess atlases in dementia research (Elsheikh et al., 2021; Ibañez et al., 2021a, 2021b; Lee et al., 2018; Liu et al., 2012; Lord et al., 2016; Reyes et al., 2018; Saba et al., 2019; Sedeño et al., 2016, 2017), future research may compare classification performance across different brain parcellations in the dementia population. As a starting point, we compared the AAL and the HCP atlas (Glasser et al., 2016) parcellation on a representative subsample (graph connectivity and graph multifeature in both modalities) and we did not find significant differences in the AUC across groups. Moreover, the pathophysiological profile evidenced in our feature importance analysis was similar. Models using both atlases prioritized hallmark-affected areas for FTD, such as inferior and dorsolateral prefrontal cortex, anterior cingulum, middle, and superior temporal areas (Filippi et al., 2013; Meeter et al., 2017; Whitwell, 2019; Whitwell et al., 2011b; Zhang et al., 2009). Furthermore, the AAL’s models prioritized also other areas with extensive evidence of affectation in FTD such as the insula and precuneus (Agosta et al., 2013; Baez et al., 2019; Popal et al., 2020; Reyes et al., 2018; Whitwell et al., 2011b). The selection of parcellation must rely on quantifiable factors such as reproducibility, clustering validity metrics, multimodal comparisons, and network analysis (Arslan et al., 2018). Importantly, no single parcellation consistently outperforms the others across all evaluation criteria (Arslan et al., 2018). Thus, by considering the various studies that used the AAL atlas in dementia research, even with ML methods (Asim et al., 2018; Bachli et al., 2020; Castellazzi et al., 2020; Hao et al., 2020; McMillan et al., 2014; Park et al., 2022; Sedeño et al., 2017), our choice of parcellation was based on the reproducibility criteria. A systematic comparison of multiple atlases across the different modalities is beyond the scope of this work. Finally, most of the patients in this study were in the early to moderate stages of the disease. Future longitudinal studies may determine the value of our approach for monitoring disease progression.

### Conclusions

We developed a multiclass characterization of FTD variants combining hundreds of functional and structural network features, as well as demographic and cognitive variables. In contrast to previous studies, we optimized the variable space by eliminating uninformative features resulting in a highly accurate FTD variant characterization. This approach can help in the future development of clinical decision support tools aimed at detecting specific affectations in the context of overlapping neurodegenerative diseases.

## ACKNOWLEDGMENTS

We thankfully acknowledge the participation of patients and controls, as well as the support of the patients’ families. Data used in preparation of this article were obtained from the Frontotemporal Lobar Degeneration Neuroimaging Initiative (FTLDNI) database (https://4rtni-ftldni.ini.usc.edu/). The investigators at NIFD/FTLDNI contributed to the design and implementation of FTLDNI and/or provided data but did not participate in analysis or writing of this report.

## SUPPORTING INFORMATION

Supporting information for this article is available at https://doi.org/10.1162/netn_a_00285. Datasets are available in their own online repository: Neuroimaging In Frontotemporal Dementia (NIFD/LONI). The code for the data analysis of this study is available from the corresponding author on reasonable request.

## AUTHOR CONTRIBUTIONS

Sebastian Moguilner: Conceptualization; Formal analysis; Investigation; Methodology; Software; Supervision; Visualization; Writing – original draft; Writing – review & editing. Raul Gonzalez-Gomez: Conceptualization; Data curation; Formal analysis; Investigation; Software; Validation; Visualization; Writing – original draft; Writing – review & editing. Agustín Ibañez: Conceptualization; Funding acquisition; Methodology; Supervision; Writing – original draft; Writing – review & editing.

## FUNDING INFORMATION

Agustín Ibáñez, Takeda Pharmaceutical Company (https://dx.doi.org/10.13039/100008373), Award ID: CW2680521. This work is partially supported by grants from CONICET; ANID/FONDECYT Regular (1170010); FONCYT-PICT 2017-1820; Sistema General de Regalías (BPIN2018000100059), Universidad del Valle (CI 5316); Alzheimer’s Association GBHI ALZ UK-20-639295; and the Multi-Partner Consortium To Expand Dementia Research In Latin America: ReDLat, supported by National Institutes of Health, National Institutes of Aging (R01 AG057234), Alzheimer’s Association (SG-20-725707), Rainwater Charitable foundation - Tau Consortium, and Global Brain Health Institute).

## TECHNICAL TERMS

Multiclass classification: Classifying positive class label subjects against a negative class comprising several categories.
eXtreme Gradient Boosting: An optimized distributed library that implements Gradient Boosting machine learning algorithms providing parallel tree boosting.
Multifeature: Combining neuroimaging features with cognitive features.
Raw connectivity: Brain connectivity analyzed at the voxel’s BOLD time series level in the case of functional connectivity, and at the tract level for structural connectivity.
Multimodal: Combining several neuroimaging methods.
Microaverage AUC: Micro-average metrics aggregate the contributions of all classes to compute the average AUC metric. This method is preferred when class imbalance is present.

## References

[bib1] Agosta, F., Galantucci, S., Valsasina, P., Canu, E., Meani, A., Marcone, A., … Filippi, M. (2014). Disrupted brain connectome in semantic variant of primary progressive aphasia. Neurobiology of Aging, 35, 2646–2655. 10.1016/j.neurobiolaging.2014.05.017, 24970567

[bib2] Agosta, F., Sala, S., Valsasina, P., Meani, A., Canu, E., Magnani, G., … Filippi, M. (2013). Brain network connectivity assessed using graph theory in frontotemporal dementia. Neurology, 81, 134–143. 10.1212/WNL.0b013e31829a33f8, 23719145

[bib3] Agosta, F., Scola, E., Canu, E., Marcone, A., Magnani, G., Sarro, L., … Filippi, M. (2012). White matter damage in frontotemporal lobar degeneration spectrum. Cerebral Cortex, 22, 2705–2714. 10.1093/cercor/bhr288, 21988828

[bib4] Armstrong, M. J., Litvan, I., Lang, A. E., Bak, T. H., Bhatia, K. P., Borroni, B., … Weiner, W. J. (2013). Criteria for the diagnosis of corticobasal degeneration. Neurology, 80, 496–503. 10.1212/WNL.0b013e31827f0fd1, 23359374 PMC3590050

[bib5] Arslan, S., Ktena, S. I., Makropoulos, A., Robinson, E. C., Rueckert, D., & Parisot, S. (2018). Human brain mapping: A systematic comparison of parcellation methods for the human cerebral cortex. NeuroImage, 170, 5–30. 10.1016/j.neuroimage.2017.04.014, 28412442

[bib6] Asim, Y., Raza, B., Malik, A. K., Rathore, S., Hussain, L., & Iftikhar, M. A. (2018). A multi-modal, multi-atlas-based approach for Alzheimer detection via machine learning. International Journal of Imaging Systems and Technology, 28(2), 113–123. 10.1002/ima.22263

[bib7] Bachli, M. B., Sedeño, L., Ochab, J. K., Piguet, O., Kumfor, F., Reyes, P., … Chialvo, D. R. (2020). Evaluating the reliability of neurocognitive biomarkers of neurodegenerative diseases across countries: A machine learning approach. NeuroImage, 208, 116456. 10.1016/j.neuroimage.2019.116456, 31841681 PMC7008715

[bib8] Baez, S., Pinasco, C., Roca, M., Ferrari, J., Couto, B., García-Cordero, I., … Torralva, T. (2019). Brain structural correlates of executive and social cognition profiles in behavioral variant frontotemporal dementia and elderly bipolar disorder. Neuropsychologia, 126, 159–169. 10.1016/j.neuropsychologia.2017.02.012, 28219620

[bib9] Basser, P. J., Mattiello, J., & LeBihan, D. (1994a). Estimation of the effective self-diffusion tensor from the NMR spin echo. Journal of Magnetic Resonance, Series B, 103, 247–254. 10.1006/jmrb.1994.1037, 8019776

[bib10] Basser, P. J., Mattiello, J., & LeBihan, D. (1994b). MR diffusion tensor spectroscopy and imaging. Biophysical Journal, 66, 259–267. 10.1016/S0006-3495(94)80775-1, 8130344 PMC1275686

[bib11] Bassett, D. S., & Bullmore, E. (2006). Small-world brain networks. Neuroscientist, 12, 512–523. 10.1177/1073858406293182, 17079517

[bib12] Behravan, H., Hartikainen, J. M., Tengström, M., Pylkäs, K., Winqvist, R., Kosma, V. M., & Mannermaa, A. (2018). Machine learning identifies interacting genetic variants contributing to breast cancer risk: A case study in Finnish cases and controls. Scientific Reports, 8, 13149. 10.1038/s41598-018-31573-5, 30177847 PMC6120908

[bib13] Bejanin, A., Tammewar, G., Marx, G., Cobigo, Y., Iaccarino, L., Kornak, J., … Rabinovici, G. D. (2020). Longitudinal structural and metabolic changes in frontotemporal dementia. Neurology, 95(2), e140–e154. 10.1212/WNL.0000000000009760, 32591470 PMC7455324

[bib14] Bharti, K., Bologna, M., Upadhyay, N., Piattella, M. C., Suppa, A., Petsas, N., … Pantano, P. (2017). Abnormal resting-state functional connectivity in progressive supranuclear palsy and corticobasal syndrome. Frontiers in Neurology, 8, 248. 10.3389/fneur.2017.00248, 28634465 PMC5459910

[bib15] Bisenius, S., Mueller, K., Diehl-Schmid, J., Fassbender, K., Grimmer, T., Jessen, F., … Schroeter, M. L. (2017). Predicting primary progressive aphasias with support vector machine approaches in structural MRI data. NeuroImage: Clinical, 14, 334–343. 10.1016/j.nicl.2017.02.003, 28229040 PMC5310935

[bib16] Boeve, B. F., Boxer, A. L., Kumfor, F., Pijnenburg, Y., & Rohrer, J. D. (2022). Advances and controversies in frontotemporal dementia: Diagnosis, biomarkers, and therapeutic considerations. The Lancet Neurology, 21(3), 258–272. 10.1016/S1474-4422(21)00341-0, 35182511

[bib17] Borroni, B., Benussi, A., Pilotto, A., Gazzina, S., Turrone, R., Gardoni, F., … Padovani, A. (2014). Diagnosing progressive supranuclear palsy: Role of biological and neuroimaging markers. Journal of Alzheimer’s Disease & Parkinsonism, 4, 168. 10.4172/2161-0460.1000168

[bib18] Boxer, A. L., Geschwind, M. D., Belfor, N., Gorno-Tempini, M. L., Schauer, G. F., Miller, B. L., … Rosen, H. J. (2006). Patterns of brain atrophy that differentiate corticobasal degeneration syndrome from progressive supranuclear palsy. Archives of Neurology, 63, 81–86. 10.1001/archneur.63.1.81, 16401739

[bib19] Bressler, S. L., & Menon, V. (2010). Large-scale brain networks in cognition: Emerging methods and principles. Trends in Cognitive Sciences, 14, 277–290. 10.1016/j.tics.2010.04.004, 20493761

[bib20] Bullmore, E., & Sporns, O. (2009). Complex brain networks: Graph theoretical analysis of structural and functional systems. Nature Reviews Neuroscience, 10, 186–198. 10.1038/nrn2575, 19190637

[bib21] Bzdok, D., Altman, N., & Krzywinski, M. (2018). Statistics versus machine learning. Nature Methods, 15, 233–234. 10.1038/nmeth.4642, 30100822 PMC6082636

[bib22] Bzdok, D., Krzywinski, M., & Altman, N. (2017). Machine learning: A primer. Nature Methods, 14, 1119–1120. 10.1038/nmeth.4526, 29664466 PMC5905345

[bib23] Callaghan, P. T. (1994). Principles of nuclear magnetic resonance microscopy. Oxford, UK: Clarendon Press.

[bib24] Castellazzi, G., Cuzzoni, M. G., Cotta Ramusino, M., Martinelli, D., Denaro, F., Ricciardi, A., … Gandini Wheeler-Kingshott, C. A. M. (2020). A machine learning approach for the differential diagnosis of Alzheimer and vascular dementia fed by MRI selected features. Frontiers in Neuroinformatics, 14, 25. 10.3389/fninf.2020.00025, 32595465 PMC7300291

[bib25] Chen, X., Lu, B., & Yan, C. G. (2018). Reproducibility of R-fMRI metrics on the impact of different strategies for multiple comparison correction and sample sizes. Human Brain Mapping, 39, 300–318. 10.1002/hbm.23843, 29024299 PMC6866539

[bib26] Chen, Y., Landin-Romero, R., Kumfor, F., Irish, M., Hodges, J. R., & Piguet, O. (2020). Cerebellar structural connectivity and contributions to cognition in frontotemporal dementias. Cortex, 129, 57–67. 10.1016/j.cortex.2020.04.013, 32428762

[bib27] Churcher, A., Ullah, R., Ahmad, J., Ur Rehman, S., Masood, F., Gogate, M., Alqahtani, F., Nour, B., & Buchanan, W. J. (2021). An experimental analysis of attack classification using machine learning in IoT networks. Sensors, 21, 446. 10.3390/s21020446, 33435202 PMC7827441

[bib28] Cohen, J. (1988). Statistical power analysis for the behavioral sciences. New York, NY: Routledge. 10.4324/9780203771587

[bib29] Cohen, J. (1992). A power primer. Psychological Bulletin, 112, 155–159. 10.1037/0033-2909.112.1.155, 19565683

[bib30] Cohen, J. D., Daw, N., Engelhardt, B., Hasson, U., Li, K., Niv, Y., … Willke, T. L. (2017). Computational approaches to fMRI analysis. Nature Neuroscience, 20, 304–313. 10.1038/nn.4499, 28230848 PMC5457304

[bib31] Custodio, N., Herrera-Perez, E., Lira, D., Roca, M., Manes, F., Báez, S., & Torralva, T. (2016). Evaluation of the INECO frontal screening and the frontal assessment battery in Peruvian patients with Alzheimer’s disease and behavioral variant frontotemporal dementia. eNeurologicalSci, 5, 25–29. 10.1016/j.ensci.2016.11.001, 29430554 PMC5803087

[bib32] Daianu, M., Mendez, M. F., Baboyan, V. G., Jin, Y., Melrose, R. J., Jimenez, E. E., & Thompson, P. M. (2016). An advanced white matter tract analysis in frontotemporal dementia and early-onset Alzheimer’s disease. Brain Imaging and Behavior, 10, 1038–1053. 10.1007/s11682-015-9458-5, 26515192 PMC5167220

[bib33] Deshpande, G., Laconte, S., Peltier, S., & Hu, X. (2009). Integrated local correlation: A new measure of local coherence in fMRI data. Human Brain Mapping, 23, 13–23. 10.1002/hbm.20482, 17979117 PMC6870773

[bib34] Dickerson, B. C., Fenstermacher, E., Salat, D. H., Wolk, D. A., Maguire, R. P., Desikan, R., … Fischl, B. (2008). Detection of cortical thickness correlates of cognitive performance: Reliability across MRI scan sessions, scanners, and field strengths. NeuroImage, 39, 10–18. 10.1016/j.neuroimage.2007.08.042, 17942325 PMC2141650

[bib35] Donnelly-Kehoe, P. A., Pascariello, G. O., & Gómez, J. C. (2018). Looking for Alzheimer’s disease morphometric signatures using machine learning techniques. Journal of Neuroscience Methods, 302, 24–34. 10.1016/j.jneumeth.2017.11.013, 29174020

[bib36] Dopper, E. G. P., Rombouts, S. A. R. B., Jiskoot, L. C., den Heijer, T., de Graaf, J. R. A., de Koning, I., … van Swieten, J. C. (2014). Structural and functional brain connectivity in presymptomatic familial frontotemporal dementia. Neurology, 83(2), e19–e26. 10.1212/WNL.0000000000000583, 25002573

[bib37] Dottori, M., Sedenõ, L., Martorell Caro, M., Alifano, F., Hesse, E., Mikulan, E., … Ibañez, A. (2017). Towards affordable biomarkers of frontotemporal dementia: A classification study via network’s information sharing. Scientific Reports, 7, 3822. 10.1038/s41598-017-04204-8, 28630492 PMC5476568

[bib38] Elsheikh, S. S. M., Chimusa, E. R., Mulder, N. J., & Crimi, A. (2021). Relating global and local connectome changes to dementia and targeted gene expression in Alzheimer’s disease. Frontiers in Human Neuroscience, 15, 761424. 10.3389/fnhum.2021.761424, 35002653 PMC8734427

[bib39] Fawcett, T. (2006). An introduction to ROC analysis. Pattern Recognition Letters, 27, 861–874. 10.1016/j.patrec.2005.10.010

[bib40] Feis, R. A., Bouts, M. J. R. J., Panman, J. L., Jiskoot, L. C., Dopper, E. G. P., Schouten, T. M., … Rombouts, S. A. R. B. (2018). Single-subject classification of presymptomatic frontotemporal dementia mutation carriers using multimodal MRI. NeuroImage: Clinical, 20, 188–196. 10.1016/j.nicl.2018.07.014, 30094168 PMC6072645

[bib41] Feurer, M., & Hutter, F. (2019). Hyperparameter optimization. In Automated machine learning: Methods, systems, challenges (pp. 3–33). Cham, Switzerland: Springer International Publishing. 10.1007/978-3-030-05318-5_1

[bib42] Filippi, M., Agosta, F., Scola, E., Canu, E., Magnani, G., Marcone, A., … Falini, A. (2013). Functional network connectivity in the behavioral variant of frontotemporal dementia. Cortex, 49, 2389–2401. 10.1016/j.cortex.2012.09.017, 23164495

[bib43] Filippi, M., Basaia, S., Canu, E., Imperiale, F., Meani, A., Caso, F., … Agosta, F. (2017). Brain network connectivity differs in early-onset neurodegenerative dementia. Neurology, 89, 1764–1772. 10.1212/WNL.0000000000004577, 28954876 PMC5664301

[bib44] Finn, E. S., Shen, X., Scheinost, D., Rosenberg, M. D., Huang, J., Chun, M. M., … Constable, R. T. (2015). Functional connectome fingerprinting: Identifying individuals using patterns of brain connectivity. Nature Neuroscience, 18, 1664–1671. 10.1038/nn.4135, 26457551 PMC5008686

[bib45] Fittipaldi, S., Abrevaya, S., de la Fuente, A., Pascariello, G. O., Hesse, E., Birba, A., … Ibáñez, A. (2020). A multidimensional and multi-feature framework for cardiac interoception. NeuroImage, 212, 116677. 10.1016/j.neuroimage.2020.116677, 32101777 PMC7165068

[bib46] Fox, M. D., & Raichle, M. E. (2007). Spontaneous fluctuations in brain activity observed with functional magnetic resonance imaging. Nature Reviews Neuroscience, 8, 700–711. 10.1038/nrn2201, 17704812

[bib47] Fox, R. J., Sakaie, K., Lee, J. C., Debbins, J. P., Liu, Y., Arnold, D. L., … Fisher, E. (2012). A validation study of multicenter diffusion tensor imaging: Reliability of fractional anisotropy and diffusivity values. American Journal of Neuroradiology, 33, 695–700. 10.3174/ajnr.A2844, 22173748 PMC8050446

[bib48] Gao, X., He, Y., Zhang, M., Diao, X., Jing, X., Ren, B., & Ji, W. (2021). A multiclass classification using one-versus-all approach with the differential partition sampling ensemble. Engineering Applications of Artificial Intelligence, 97, 104034. 10.1016/j.engappai.2020.104034

[bib49] Glasser, M. F., Coalson, T. S., Robinson, E. C., Hacker, C. D., Harwell, J., Yacoub, E., … van Essen, D. C. (2016). A multi-modal parcellation of human cerebral cortex. Nature, 536(7615), 171–178. 10.1038/nature18933, 27437579 PMC4990127

[bib50] Gonzalez Campo, C., Salamone, P., Rodriguez-Arriagada, N., Richter, F., Herrera, E., Bruno, D., … Sedeno, L. (2020). Fatigue in multiple sclerosis is associated to multimodal interoceptive abnormalities. Multiple Sclerosis Journal, 26(14), 1845–1853. 10.1177/1352458519888881, 31778101 PMC7253315

[bib51] Gorno-Tempini, M. L., Hillis, A. E., Weintraub, S., Kertesz, A., Mendez, M., Cappa, S. F., … Grossman, M. (2011). Classification of primary progressive aphasia and its variants. Neurology, 76, 1006–1014. 10.1212/WNL.0b013e31821103e6, 21325651 PMC3059138

[bib52] Hafkemeijer, A., Möller, C., Dopper, E. G. P., Jiskoot, L. C., van den Berg-Huysmans, A. A., van Swieten, J. C., … Rombouts, S. A. R. B. (2017). A longitudinal study on resting state functional connectivity in behavioral variant frontotemporal dementia and Alzheimer’s disease. Journal of Alzheimer’s Disease, 55, 521–537. 10.3233/JAD-150695, 27662284

[bib53] Han, X., Jovicich, J., Salat, D., van der Kouwe, A., Quinn, B., Czanner, S., … Fischl, B. (2006). Reliability of MRI-derived measurements of human cerebral cortical thickness: The effects of field strength, scanner upgrade and manufacturer. NeuroImage, 32, 180–194. 10.1016/j.neuroimage.2006.02.051, 16651008

[bib54] Hao, X., Bao, Y., Guo, Y., Yu, M., Zhang, D., Risacher, S. L., … Shen, L. (2020). Multi-modal neuroimaging feature selection with consistent metric constraint for diagnosis of Alzheimer’s disease. Medical Image Analysis, 60, 101625. 10.1016/j.media.2019.101625, 31841947 PMC6980345

[bib55] Hecke, W. V., Emsell, L., & Sunaert, S. (2016). Diffusion tensor imaging: A practical handbook. Cham, Switzerland: Springer. 10.1007/978-1-4939-3118-7

[bib56] Hohenfeld, C., Werner, C. J., & Reetz, K. (2018). Resting-state connectivity in neurodegenerative disorders: Is there potential for an imaging biomarker? NeuroImage: Clinical, 18, 849–870. 10.1016/j.nicl.2018.03.013, 29876270 PMC5988031

[bib57] Hou, T., Bian, Y., McGuire, T., & Xie, X.-Q. (2021). Integrated multi-class classification and prediction of GPCR allosteric modulators by machine learning intelligence. Biomolecules, 11(6), 870. 10.3390/biom11060870, 34208096 PMC8230833

[bib58] Huys, Q. J. M., Maia, T. V., & Frank, M. J. (2016). Computational psychiatry as a bridge from neuroscience to clinical applications. Nature Neuroscience, 19, 404–413. 10.1038/nn.4238, 26906507 PMC5443409

[bib59] Iaccarino, L., Crespi, C., Della Rosa, P. A., Catricalà, E., Guidi, L., Marcone, A., … Perani, D. (2015). The semantic variant of primary progressive aphasia: Clinical and neuroimaging evidence in single subjects. PLoS One, 10, e0120197. 10.1371/journal.pone.0120197, 25756991 PMC4354903

[bib60] Ibañez, A., Fittipaldi, S., Trujillo, C., Jaramillo, T., Torres, A., Cardona, J. F., … Baez, S. (2021a). Predicting and characterizing neurodegenerative subtypes with multimodal neurocognitive signatures of social and cognitive processes. Journal of Alzheimer’s Disease, 83, 227–248. 10.3233/JAD-210163, 34275897 PMC8461708

[bib61] Ibañez, A., Yokoyama, J. S., Possin, K. L., Matallana, D., Lopera, F., Nitrini, R., Takada, L. T., Custodio, N., Sosa Ortiz, A. L., Avila-Funes, J. A., Behrens, M. I., Slachevsky, A., Myers, R. M., Cochran, J. N., Brusco, L. I., Bruno, M. A., Brucki, S. M. D., Pina-Escudero, S. D., Okada de Oliveira, M., … Miller, B. L. (2021b). The Multi-Partner Consortium to Expand Dementia Research in Latin America (ReDLat): Driving multicentric research and implementation science. Frontiers in Neurology, 12, 631722. 10.3389/fneur.2021.631722, 33776890 PMC7992978

[bib62] Jalilianhasanpour, R., Beheshtian, E., Sherbaf, G., Sahraian, S., & Sair, H. I. (2019). Functional connectivity in neurodegenerative disorders: Alzheimer’s disease and frontotemporal dementia. Topics in Magnetic Resonance Imaging, 28, 317–324. 10.1097/RMR.0000000000000223, 31794504

[bib63] Jastorff, J., De Winter, F.-L., Van den Stock, J., Vandenberghe, R., Giese, M. A., & Vandenbulcke, M. (2016). Functional dissociation between anterior temporal lobe and inferior frontal gyrus in the processing of dynamic body expressions: Insights from behavioral variant frontotemporal dementia. Human Brain Mapping, 37, 4472–4486. 10.1002/hbm.23322, 27510944 PMC6867423

[bib64] Kassraian-Fard, P., Matthis, C., Balsters, J. H., Maathuis, M. H., & Wenderoth, N. (2016). Promises, pitfalls, and basic guidelines for applying machine learning classifiers to psychiatric imaging data, with autism as an example. Frontiers in Psychiatry, 7, 177. 10.3389/fpsyt.2016.00177, 27990125 PMC5133050

[bib65] Kaufmann, T., van der Meer, D., Doan, N. T., Schwarz, E., Lund, M. J., Agartz, I., … Westlye, L. T. (2019). Common brain disorders are associated with heritable patterns of apparent aging of the brain. Nature Neuroscience, 22, 1617–1623. 10.1038/s41593-019-0471-7, 31551603 PMC6823048

[bib66] Khazaee, A., Ebrahimzadeh, A., & Babajani-Feremi, A. (2015). Identifying patients with Alzheimer’s disease using resting-state fMRI and graph theory. Clinical Neurophysiology, 126, 2132–2141. 10.1016/j.clinph.2015.02.060, 25907414

[bib67] Kim, J. P., Kim, J., Park, Y. H., Park, S. B., Lee, J. S., Yoo, S., … Seong, J. K. (2019). Machine learning based hierarchical classification of frontotemporal dementia and Alzheimer’s disease. NeuroImage: Clinical, 23, 101811. 10.1016/j.nicl.2019.101811, 30981204 PMC6458431

[bib68] Koyejo, O., Natarajan, N., Ravikumar, P., & Dhillon, I. S. (2015). Advances in neural information processing systems. Paper presented at the Proceedings of the 28th International Conference on Neural Information Processing Systems.

[bib69] Kramer, A. O., Alioto, A. G., & Kramer, J. H. (2020). Neurodegenerative conditions: FTD. In K. Sweeny, M. L. Robbins, & L. M. Cohen (Eds.), The Wiley encyclopedia of health psychology (1st ed., pp. 209–218). John Wiley & Sons. 10.1002/9781119057840.ch25

[bib70] Lee, W. J., Han, C. E., Aganj, I., Seo, S. W., & Seong, J. K. (2018). Distinct patterns of rich club organization in Alzheimer’s disease and subcortical vascular dementia: A white matter network study. Journal of Alzheimer’s Disease, 63(3), 977–987. 10.3233/JAD-180027, 29710719

[bib71] Liu, Z., Zhang, Y., Yan, H., Bai, L., Dai, R., Wei, W., … Tian, J. (2012). Altered topological patterns of brain networks in mild cognitive impairment and Alzheimer’s disease: A resting-state fMRI study. Psychiatry Research: Neuroimaging, 202(2), 118–125. 10.1016/j.pscychresns.2012.03.002, 22695315

[bib72] Lord, A., Ehrlich, S., Borchardt, V., Geisler, D., Seidel, M., Huber, S., … Walter, M. (2016). Brain parcellation choice affects disease-related topology differences increasingly from global to local network levels. Psychiatry Research: Neuroimaging, 249, 12–19. 10.1016/j.pscychresns.2016.02.001, 27000302

[bib73] Lu, P. H., Mendez, M. F., Lee, G. J., Leow, A. D., Lee, H.-W., Shapira, J., … Knopman, D. S. (2013). Patterns of brain atrophy in clinical variants of frontotemporal lobar degeneration. Dementia and Geriatric Cognitive Disorders, 35, 34–50. 10.1159/000345523, 23306166 PMC3609420

[bib74] Ma, D., Lu, D., Popuri, K., Wang, L., & Beg, M. F. (2020). Differential diagnosis of frontotemporal dementia, Alzheimer’s disease, and normal aging using a multi-scale multi-type feature generative adversarial deep neural network on structural magnetic resonance images. Frontiers in Neuroscience, 14, 853. 10.3389/fnins.2020.00853, 33192235 PMC7643018

[bib75] Mahoney, C. J., Ridgway, G. R., Malone, I. B., Downey, L. E., Beck, J., Kinnunen, K. M., … Warren, J. D. (2014). Profiles of white matter tract pathology in frontotemporal dementia. Human Brain Mapping, 35, 4163–4179. 10.1002/hbm.22468, 24510641 PMC4312919

[bib76] Mahoney, C. J., Simpson, I. J. A., Nicholas, J. M., Fletcher, P. D., Downey, L. E., Golden, H. L., … Fox, N. C. (2015). Longitudinal diffusion tensor imaging in frontotemporal dementia. Annals of Neurology, 77, 33–46. 10.1002/ana.24296, 25363208 PMC4305215

[bib77] Maier-Hein, K. H., Neher, P. F., Houde, J.-C., Côté, M.-A., Garyfallidis, E., Zhong, J., … Descoteaux, M. (2017). The challenge of mapping the human connectome based on diffusion tractography. Nature Communications, 8, 1349. 10.1038/s41467-017-01285-x, 29116093 PMC5677006

[bib78] Makridakis, S., Spiliotis, E., & Assimakopoulos, V. (2018). Statistical and machine learning forecasting methods: Concerns and ways forward. PLoS One, 13(3), e0194889. 10.1371/journal.pone.0194889, 29584784 PMC5870978

[bib79] Mandelli, M. L., Vilaplana, E., Brown, J. A., Hubbard, H. I., Binney, R. J., Attygalle, S., … Gorno-Tempini, M. L. (2016). Healthy brain connectivity predicts atrophy progression in non-fluent variant of primary progressive aphasia. Brain, 139, 2778–2791. 10.1093/brain/aww195, 27497488 PMC5035819

[bib80] Mandelli, M. L., Welch, A. E., Vilaplana, E., Watson, C., Battistella, G., Brown, J. A., … Gorno-Tempini, M. L. (2018). Altered topology of the functional speech production network in non-fluent/agrammatic variant of PPA. Cortex, 108, 252–264. 10.1016/j.cortex.2018.08.002, 30292076 PMC6317366

[bib81] Manera, A. L., Dadar, M., Collins, D. L., & Ducharme, S. (2019). Deformation based morphometry study of longitudinal MRI changes in behavioral variant frontotemporal dementia. NeuroImage: Clinical, 24, 102079. 10.1016/j.nicl.2019.102079, 31795051 PMC6879994

[bib82] Mason, L., Baxter, J., Bartlett, P., & Frean, M. (1999). Boosting algorithms as gradient descent in function space. Advances in Neural Information Processing, 12, 512–518.

[bib83] McMillan, C. T., Avants, B. B., Cook, P., Ungar, L., Trojanowski, J. Q., & Grossman, M. (2014). The power of neuroimaging biomarkers for screening frontotemporal dementia. Human Brain Mapping, 35, 4827–4840. 10.1002/hbm.22515, 24687814 PMC4107021

[bib84] McMillan, C. T., Brun, C., Siddiqui, S., Churgin, M., Libon, D., Yushkevich, P., … Grossman, M. (2012). White matter imaging contributes to the multimodal diagnosis of frontotemporal lobar degeneration. Neurology, 78, 1761–1768. 10.1212/WNL.0b013e31825830bd, 22592372 PMC3359585

[bib85] Meeter, L. H., Kaat, L. D., Rohrer, J. D., & van Swieten, J. C. (2017). Imaging and fluid biomarkers in frontotemporal dementia. Nature Reviews Neurology, 13, 406–419. 10.1038/nrneurol.2017.75, 28621768

[bib86] Meijboom, R., Steketee, R. M. E., Ham, L. S., van der Lugt, A., van Swieten, J. C., & Smits, M. (2017). Differential hemispheric predilection of microstructural white matter and functional connectivity abnormalities between respectively semantic and behavioral variant frontotemporal dementia. Journal of Alzheimer’s Disease, 56(2), 789–804. 10.3233/JAD-160564, 28059782

[bib87] Melzer, T. R., Keenan, R. J., Leeper, G. J., Kingston-Smith, S., Felton, S. A., Green, S. K., … Myall, D. J. (2020). Test-retest reliability and sample size estimates after MRI scanner relocation. NeuroImage, 211, 116608. 10.1016/j.neuroimage.2020.116608, 32032737

[bib88] Meyer, S., Mueller, K., Stuke, K., Bisenius, S., Diehl-Schmid, J., Jessen, F., … Schroeter, M. L. (2017). Predicting behavioral variant frontotemporal dementia with pattern classification in multi-center structural MRI data. NeuroImage: Clinical, 14, 656–662. 10.1016/j.nicl.2017.02.001, 28348957 PMC5357695

[bib89] Moguilner, S., García, A. M., Perl, Y. S., Tagliazucchi, E., Piguet, O., Kumfor, F., … Ibáñez, A. (2021). Dynamic brain fluctuations outperform connectivity measures and mirror pathophysiological profiles across dementia subtypes: A multicenter study. NeuroImage, 225, 117522. 10.1016/j.neuroimage.2020.117522, 33144220 PMC7832160

[bib90] Mohanty, R., Sethares, W. A., Nair, V. A., & Prabhakaran, V. (2020). Rethinking measures of functional connectivity via feature extraction. Scientific Reports, 10, 1298. 10.1038/s41598-020-57915-w, 31992762 PMC6987226

[bib91] Möller, C., Hafkemeijer, A., Pijnenburg, Y. A. L., Rombouts, S. A. R. B., van der Grond, J., Dopper, E., … van der Flier, W. M. (2015). Joint assessment of white matter integrity, cortical and subcortical atrophy to distinguish AD from behavioral variant FTD: A two-center study. NeuroImage: Clinical, 9, 418–429. 10.1016/j.nicl.2015.08.022, 26594624 PMC4600847

[bib92] Montavon, G., Samek, W., & Müller, K.-R. (2018). Methods for interpreting and understanding deep neural networks. Digital Signal Processing, 73, 1–15. 10.1016/j.dsp.2017.10.011

[bib93] Moral-Rubio, C., Balugo, P., Fraile-Pereda, A., Pytel, V., Fernández-Romero, L., Delgado-Alonso, C., … Ayala, J. L. (2021). Application of machine learning to electroencephalography for the diagnosis of primary progressive aphasia: A pilot study. Brain Sciences, 11, 1262. 10.3390/brainsci11101262, 34679327 PMC8534262

[bib94] Mori, S., & van Zijl, P. C. M. (2002). Fiber tracking: Principles and strategies—A technical review. NMR in Biomedicine, 15, 468–480. 10.1002/nbm.781, 12489096

[bib95] Mueller, K., Mildner, T., Fritz, T., Lepsien, J., Schwarzbauer, C., Schroeter, M. L., & Möller, H. E. (2011). Investigating brain response to music: A comparison of different fMRI acquisition schemes. NeuroImage, 54, 337–343. 10.1016/j.neuroimage.2010.08.029, 20728550

[bib96] Müller, A. C., & Guido, S. (2016). Introduction to machine learning with Python: A guide for data scientists. Sebastopol, CA: O’Reilly Media.

[bib97] Nguyen, T., Bertoux, M., O’Callaghan, C., Ahmed, S., Hodges, J. R., & Hornberger, M. (2013). Grey and white matter brain network changes in frontotemporal dementia subtypes. Translational Neuroscience, 4, 410–418. 10.2478/s13380-013-0141-2

[bib98] Nicholls, H. L., John, C. R., Watson, D. S., Munroe, P. B., Barnes, M. R., & Cabrera, C. P. (2020). Reaching the end-game for GWAS: Machine learning approaches for the prioritization of complex disease loci. Frontiers in Genetics, 11, 350. 10.3389/fgene.2020.00350, 32351543 PMC7174742

[bib99] Nieto-Castanon, A. (2020). Handbook of functional connectivity magnetic resonance imaging methods in CONN. Boston, MA: Hilbert Press. 10.56441/hilbertpress.2207.6598

[bib100] Noble, S., Scheinost, D., Finn, E. S., Shen, X., Papademetris, X., McEwen, S. C., … Constable, R. T. (2017a). Multisite reliability of MR-based functional connectivity. NeuroImage, 146, 959–970. 10.1016/j.neuroimage.2016.10.020, 27746386 PMC5322153

[bib101] Noble, S., Spann, M. N., Tokoglu, F., Shen, X., Constable, R. T., & Scheinost, D. (2017b). Influences on the test-retest reliability of functional connectivity MRI and its relationship with behavioral utility. Cerebral Cortex, 27, 5415–5429. 10.1093/cercor/bhx230, 28968754 PMC6248395

[bib102] Oishi, K., Zilles, K., Amunts, K., Faria, A., Jiang, H., Li, X., … Mori, S. (2008). Human brain white matter atlas: Identification and assignment of common anatomical structures in superficial white matter. NeuroImage, 43, 447–457. 10.1016/j.neuroimage.2008.07.009, 18692144 PMC2586008

[bib103] Olney, N. T., Spina, S., & Miller, B. L. (2017). Frontotemporal dementia. Neurologic Clinics, 35, 339–374. 10.1016/j.ncl.2017.01.008, 28410663 PMC5472209

[bib104] Ossenkoppele, R., Smith, R., Mattsson-Carlgren, N., Groot, C., Leuzy, A., Strandberg, O., … Hansson, O. (2021). Accuracy of tau positron emission tomography as a prognostic marker in preclinical and prodromal Alzheimer disease: A head-to-head comparison against amyloid positron emission tomography and magnetic resonance imaging. JAMA Neurology, 78, 961–971. 10.1001/jamaneurol.2021.1858, 34180956 PMC8240013

[bib105] Park, B., Choi, B. J., Lee, H., Jang, J. H., Roh, H. W., Kim, E. Y., … Yoon, D. (2022). Modeling brain volume using deep learning-based physical activity features in patients with dementia. Frontiers in Neuroinformatics, 16, 795171. 10.3389/fninf.2022.795171, 35356447 PMC8959707

[bib106] Peet, B. T., Spina, S., Mundada, N., & La Joie, R. (2021). Neuroimaging in frontotemporal dementia: Heterogeneity and relationships with underlying neuropathology. Neurotherapeutics, 18, 728–752. 10.1007/s13311-021-01101-x, 34389969 PMC8423978

[bib107] Pereira, F., Mitchell, T., & Botvinick, M. (2009). Machine learning classifiers and fMRI: A tutorial overview. NeuroImage, 45, S199–S209. 10.1016/j.neuroimage.2008.11.007, 19070668 PMC2892746

[bib108] Pievani, M., de Haan, W., Wu, T., Seeley, W. W., & Frisoni, G. B. (2011). Functional network disruption in the degenerative dementias. The Lancet Neurology, 10, 829–843. 10.1016/S1474-4422(11)70158-2, 21778116 PMC3219874

[bib109] Poldrack, R. A., Huckins, G., & Varoquaux, G. (2019). Establishment of best practices for evidence for prediction: A review. JAMA Psychiatry, 77, 534–540. 10.1001/jamapsychiatry.2019.3671, 31774490 PMC7250718

[bib110] Popal, H., Quimby, M., Hochberg, D., Dickerson, B. C., & Collins, J. A. (2020). Altered functional connectivity of cortical networks in semantic variant primary progressive aphasia. NeuroImage: Clinical, 28, 102494. 10.1016/j.nicl.2020.102494, 33395985 PMC7708956

[bib111] Premi, E., Cauda, F., Costa, T., Diano, M., Gazzina, S., Gualeni, V., … Borroni, B. (2016). Looking for neuroimaging markers in frontotemporal lobar degeneration clinical trials: A multi-voxel pattern analysis study in Granulin disease. Journal of Alzheimer’s Disease, 51, 249–262. 10.3233/JAD-150340, 26836150

[bib112] Rascovsky, K., & Grossman, M. (2013). Clinical diagnostic criteria and classification controversies in frontotemporal lobar degeneration. International Review of Psychiatry, 25, 145–158. 10.3109/09540261.2013.763341, 23611345 PMC3906583

[bib113] Rascovsky, K., Hodges, J. R., Knopman, D., Mendez, M. F., Kramer, J. H., Neuhaus, J., … Miller, B. L. (2011). Sensitivity of revised diagnostic criteria for the behavioural variant of frontotemporal dementia. Brain, 134, 2456–2477. 10.1093/brain/awr179, 21810890 PMC3170532

[bib114] Reyes, P., Ortega-Merchan, M. P., Rueda, A., Uriza, F., Santamaria-García, H., Rojas-Serrano, N., … Matallana, D. (2018). Functional connectivity changes in behavioral, semantic, and nonfluent variants of frontotemporal dementia. Behavioural Neurology, 2018, 9684129. 10.1155/2018/9684129, 29808100 PMC5902123

[bib115] Rohan, V., Chaney, G. A. S., Deright, J., & Onyike, C. U. (2019). A meta-analysis of neuropsychological, social cognitive, and olfactory functioning in the behavioral and language variants of frontotemporal dementia. Psychological Medicine, 49, 2669–2680. 10.1017/S0033291718003604, 30520407

[bib116] Rosenthal, J. A. (1996). Qualitative descriptors of strength of association and effect size. Journal of Social Service Research, 21, 37–59. 10.1300/J079v21n04_02

[bib117] Rubinov, M., & Sporns, O. (2010). Complex network measures of brain connectivity: Uses and interpretations. NeuroImage, 52, 1059–1069. 10.1016/j.neuroimage.2009.10.003, 19819337

[bib118] Saba, V., Premi, E., Cristillo, V., Gazzina, S., Palluzzi, F., Zanetti, O., … Grassi, M. (2019). Brain connectivity and information-flow breakdown revealed by a minimum spanning tree-based analysis of MRI data in behavioral variant frontotemporal dementia. Frontiers in Neuroscience, 13, 211. 10.3389/fnins.2019.00211, 30930736 PMC6427927

[bib119] Salvatore, C., Cerasa, A., Castiglioni, I., Gallivanone, F., Augimeri, A., Lopez, M., … Quattrone, A. (2014). Machine learning on brain MRI data for differential diagnosis of Parkinson’s disease and progressive supranuclear palsy. Journal of Neuroscience Methods, 222, 230–237. 10.1016/j.jneumeth.2013.11.016, 24286700

[bib120] Santillo, A. F., Martensson, J., Lindberg, O., Nilsson, M., Manzouri, A., Landqvist Waldö, M., … Nilsson, C. (2013). Diffusion tensor tractography versus volumetric imaging in the diagnosis of behavioral variant frontotemporal dementia. PLoS One, 8, e66932. 10.1371/journal.pone.0066932, 23874403 PMC3715470

[bib121] Schwartzman, B. A., Dougherty, R. F., & Taylor, J. E. (2008). False discovery rate analysisof brain diffusion. The Annals of Applied Statistics, 2, 153–175. 10.1214/07-AOAS133, 35388313 PMC8982959

[bib122] Sedeño, L., Couto, B., García-Cordero, I., Melloni, M., Baez, S., Sepúlveda, J. P. M., … Ibañez, A. (2016). Brain network organization and social executive performance in frontotemporal dementia. Journal of the International Neuropsychological Society, 22, 250–262. 10.1017/S1355617715000703, 26888621

[bib123] Sedeño, L., Piguet, O., Abrevaya, S., Desmaras, H., García-Cordero, I., Baez, S., … Ibañez, A. (2017). Tackling variability: A multicenter study to provide a gold-standard network approach for frontotemporal dementia. Human Brain Mapping, 38, 3804–3822. 10.1002/hbm.23627, 28474365 PMC6867023

[bib124] Seeley, W. W., Crawford, R., Rascovsky, K., Kramer, J. H., Weiner, M., Miller, B. L., & Gorno-Tempini, M. L. (2008). Frontal paralimbic network atrophy in very mild behavioral variant frontotemporal dementia. Archives of Neurology, 65, 249–255. 10.1001/archneurol.2007.38, 18268196 PMC2544627

[bib125] Seeley, W. W., Crawford, R. K., Zhou, J., Miller, B. L., & Greicius, M. D. (2009). Neurodegenerative diseases target large-scale human brain networks. Neuron, 62, 42–52. 10.1016/j.neuron.2009.03.024, 19376066 PMC2691647

[bib126] Sheelakumari, R., Bineesh, C., Varghese, T., Kesavadas, C., & Verghese, J. (2020). Neuroanatomical correlates of apathy and disinhibition in behavioural variant frontotemporal dementia. Brain Imaging and Behavior, 14, 2004–2011. 10.1007/s11682-019-00150-3, 31273672 PMC6942247

[bib127] Smith, S. M., & Nichols, T. E. (2009). Threshold-free cluster enhancement: Addressing problems of smoothing, threshold dependence and localisation in cluster inference. NeuroImage, 44, 83–98. 10.1016/j.neuroimage.2008.03.061, 18501637

[bib128] Sporns, O. (2010). Networks of the brain. Cambridge, MA: MIT Press. 10.7551/mitpress/8476.001.0001

[bib129] Sporns, O. (2018). Graph theory methods: Applications in brain networks. Dialogues in Clinical Neuroscience, 20, 111–121. 10.31887/DCNS.2018.20.2/osporns, 30250388 PMC6136126

[bib130] Staffaroni, A. M., Ljubenkov, P. A., Kornak, J., Cobigo, Y., Datta, S., Marx, G., … Rosen, H. J. (2019). Longitudinal multimodal imaging and clinical endpoints for frontotemporal dementia clinical trials. Brain, 142(2), 443–459. 10.1093/brain/awy319, 30698757 PMC6351779

[bib131] Tahmasian, M., Shao, J., Meng, C., Grimmer, T., Diehl-Schmid, J., Yousefi, B. H., … Sorg, C. (2016). Based on the network degeneration hypothesis: Separating individual patients with different neurodegenerative syndromes in a preliminary hybrid PET/MR study. Journal of Nuclear Medicine, 57, 410–415. 10.2967/jnumed.115.165464, 26585059

[bib132] Torlay, L., Perrone-Bertolotti, M., Thomas, E., & Baciu, M. (2017). Machine learning–XGBoost analysis of language networks to classify patients with epilepsy. Brain Informatics, 4, 159–169. 10.1007/s40708-017-0065-7, 28434153 PMC5563301

[bib133] Torralva, T., Roca, M., Gleichgerrcht, E., López, P., & Manes, F. (2009). INECO Frontal Screening (IFS): A brief, sensitive, and specific tool to assess executive functions in dementia. Journal of the International Neuropsychological Society, 15, 777–786. 10.1017/S1355617709990415, 19635178

[bib134] Torso, M., Bozzali, M., Cercignani, M., Jenkinson, M., & Chance, S. A. (2020). Using diffusion tensor imaging to detect cortical changes in fronto-temporal dementia subtypes. Scientific Reports, 10, 11237. 10.1038/s41598-020-68118-8, 32641807 PMC7343779

[bib135] Torso, M., Ridgway, G. R., Jenkinson, M., & Chance, S. (2021). Intracortical diffusion tensor imaging signature of microstructural changes in frontotemporal lobar degeneration. Alzheimer’s Research and Therapy, 13, 180. 10.1186/s13195-021-00914-4, 34686217 PMC8539736

[bib136] Tournier, J.-D. (2010). The biophysics of crossing fibers. In D. K. Jones (Ed.), Diffusion MRI: Theory, methods, and application (pp. 465–482). Oxford, UK: Oxford University Press. 10.1093/med/9780195369779.003.0028

[bib137] Tovar-Moll, F., de Oliveira-Souza, R., Bramati, I. E., Zahn, R., Cavanagh, A., Tierney, M., … Grafman, J. (2014). White matter tract damage in the behavioral variant of frontotemporal and corticobasal dementia syndromes. PLoS One, 9(7), e102656. 10.1371/journal.pone.0102656, 25054218 PMC4108323

[bib138] Tsai, R. M., Bejanin, A., Lesman-Segev, O., La Joie, R., Visani, A., Bourakova, V., … Rabinovici, G. D. (2019). ^18^F-flortaucipir (AV-1451) tau PET in frontotemporal dementia syndromes. Alzheimer’s Research and Therapy, 11, 13. 10.1186/s13195-019-0470-7, 30704514 PMC6357510

[bib139] Tzourio-Mazoyer, N., Landeau, B., Papathanassiou, D., Crivello, F., Etard, O., Delcroix, N., … Joliot, M. (2002). Automated anatomical labeling of activations in SPM using a macroscopic anatomical parcellation of the MNI MRI single-subject brain. NeuroImage, 15, 273–289. 10.1006/nimg.2001.0978, 11771995

[bib140] Upadhyay, N., Suppa, A., Piattella, M. C., Bologna, M., Di Stasio, F., Formica, A., … Pantano, P. (2016). MRI gray and white matter measures in progressive supranuclear palsy and corticobasal syndrome. Journal of Neurology, 263, 2022–2031. 10.1007/s00415-016-8224-y, 27411806

[bib141] van Wijk, B. C. M., Stam, C. J., & Daffertshofer, A. (2010). Comparing brain networks of different size and connectivity density using graph theory. PLoS One, 5, e13701. 10.1371/journal.pone.0013701, 21060892 PMC2965659

[bib142] Venkatraman, E. S. (2000). A permutation test to compare receiver operating characteristic curves. Biometrics, 56, 1134–1138. 10.1111/j.0006-341X.2000.01134.x, 11129471

[bib143] Veraart, J., Fieremans, E., & Novikov, D. S. (2016). Diffusion MRI noise mapping using random matrix theory. Magnetic Resonance in Medicine, 76, 1582–1593. 10.1002/mrm.26059, 26599599 PMC4879661

[bib144] Wade, C. (2020). Hands-on gradient boosting with XGBoost and scikit-learn: Perform accessible machine learning and extreme gradient boosting with Python. Birmingham, UK: Packt Publishing.

[bib145] Whitfield-Gabrieli, S., & Nieto-Castanon, A. (2012). Conn: A functional connectivity toolbox for correlated and anticorrelated brain networks. Brain Connectivity, 2, 125–141. 10.1089/brain.2012.0073, 22642651

[bib146] Whitwell, J. L. (2019). Neuroimaging across the FTD spectrum. In J. T. Becker & A. D. Cohen (Eds.), Progress in molecular biology and translational science (Vol. 165, pp. 187–223). Academic Press. 10.1016/bs.pmbts.2019.05.009, PMC7153045 31481163

[bib147] Whitwell, J. L., Avula, R., Master, A., Vemuri, P., Senjem, M. L., Jones, D. T., … Josephs, K. A. (2011a). Disrupted thalamocortical connectivity in PSP: A resting-state fMRI, DTI, and VBM study. Parkinsonism and Related Disorders, 17, 599–605. 10.1016/j.parkreldis.2011.05.013, 21665514 PMC3168952

[bib148] Whitwell, J. L., Avula, R., Senjem, M. L., Kantarci, K., Weigand, S. D., Samikoglu, A., … Jack, C. R. (2010a). Gray and white matter water diffusion in the syndromic variants of frontotemporal dementia. Neurology, 74, 1279–1287. 10.1212/WNL.0b013e3181d9edde, 20404309 PMC2860485

[bib149] Whitwell, J. L., Jack, C. R., Boeve, B. F., Parisi, J. E., Ahlskog, J. E., Drubach, D. A., … Josephs, K. A. (2010b). Imaging correlates of pathology in corticobasal syndrome. Neurology, 75, 1879–1887. 10.1212/WNL.0b013e3181feb2e8, 21098403 PMC2995388

[bib150] Whitwell, J. L., Josephs, K. A., Avula, R., Tosakulwong, N., Weigand, S. D., Senjem, M. L., … Jack, C. R. (2011b). Altered functional connectivity in asymptomatic MAPT subjects A comparison to bvFTD. Neurology, 77, 866–874. 10.1212/WNL.0b013e31822c61f2, 21849646 PMC3162637

[bib151] Whitwell, J. L., Przybelski, S. A., Weigand, S. D., Ivnik, R. J., Vemuri, P., Gunter, J. L., … Josephs, K. A. (2009). Distinct anatomical subtypes of the behavioural variant of frontotemporal dementia: A cluster analysis study. Brain, 132, 2932–2946. 10.1093/brain/awp232, 19762452 PMC2768663

[bib152] Whitwell, J. L., Schwarz, C. G., Reid, R. I., Kantarci, K., Jack, C. R., & Josephs, K. A. (2014). Diffusion tensor imaging comparison of progressive supranuclear palsy and corticobasal syndromes. Parkinsonism and Related Disorders, 20, 493–498. 10.1016/j.parkreldis.2014.01.023, 24656943

[bib153] Wilcox, R. (2017). Introduction to robust estimation and hypothesis testing. Amsterdam, The Netherlands: Elsevier.

[bib154] Wolpe, N., Moore, J. W., Rae, C. L., Rittman, T., Altena, E., Haggard, P., & Rowe, J. B. (2014). The medial frontal-prefrontal network for altered awareness and control of action in corticobasal syndrome. Brain, 137, 208–220. 10.1093/brain/awt302, 24293266 PMC3891444

[bib155] Xuan, P., Sun, C., Zhang, T., Ye, Y., Shen, T., & Dong, Y. (2019). Gradient boosting decision tree-based method for predicting interactions between target genes and drugs. Frontiers in Genetics, 10, 459. 10.3389/fgene.2019.00459, 31214240 PMC6555260

[bib156] Yang, H., Long, X. Y., Yang, Y., Yan, H., Zhu, C. Z., Zhou, X. P., … Gong, Q. Y. (2007). Amplitude of low frequency fluctuation within visual areas revealed by resting-state functional MRI. NeuroImage, 36, 144–152. 10.1016/j.neuroimage.2007.01.054, 17434757

[bib157] Yeh, F.-C., Liu, L., Hitchens, T. K., & Wu, Y. L. (2017). Mapping immune cell infiltration using restricted diffusion MRI. Magnetic Resonance in Medicine, 77, 603–612. 10.1002/mrm.26143, 26843524 PMC8052951

[bib158] Yeh, F.-C., Panesar, S., Barrios, J., Fernandes, D., Abhinav, K., Meola, A., & Fernandez-Miranda, J. C. (2019a). Automatic removal of false connections in diffusion MRI tractography using topology-informed pruning (TIP). Neurotherapeutics, 16, 52–58. 10.1007/s13311-018-0663-y, 30218214 PMC6361061

[bib159] Yeh, F.-C., Panesar, S., Fernandes, D., Meola, A., Yoshino, M., Fernandez-Miranda, J. C., … Verstynen, T. D. (2018). Population-averaged atlas of the macroscale human structural connectome and its network topology. NeuroImage, 178, 57–68. 10.1016/j.neuroimage.2018.05.027, 29758339 PMC6921501

[bib160] Yeh, F.-C., & Tseng, W.-Y. I. (2011). NTU-90: A high angular resolution brain atlas constructed by q-space diffeomorphic reconstruction. NeuroImage, 58, 91–99. 10.1016/j.neuroimage.2011.06.021, 21704171

[bib161] Yeh, F.-C., Verstynen, T. D., Wang, Y., Fernández-Miranda, J. C., & Tseng, W.-Y. I. (2013). Deterministic diffusion fiber tracking improved by quantitative anisotropy. PLoS One, 8, e80713. 10.1371/journal.pone.0080713, 24348913 PMC3858183

[bib162] Yeh, F.-C., Wedeen, V. J., & Tseng, W.-Y. I. (2010). Generalized q-sampling imaging. IEEE Transactions on Medical Imaging, 29, 1626–1635. 10.1109/TMI.2010.2045126, 20304721

[bib163] Yeh, F.-C., Zaydan, I. M., Suski, V. R., Lacomis, D., Richardson, R. M., Maroon, J. C., & Barrios-Martinez, J. (2019b). Differential tractography as a track-based biomarker for neuronal injury. NeuroImage, 202, 116131. 10.1016/j.neuroimage.2019.116131, 31472253 PMC6919327

[bib164] Younes, K., & Miller, B. L. (2020). Neuropsychiatric aspects of frontotemporal dementia. Psychiatric Clinics of North America, 43, 345–360. 10.1016/j.psc.2020.02.005, 32439026

[bib165] Yu, J., & Lee, T. M. C. (2019). The longitudinal decline of white matter microstructural integrity in behavioral variant frontotemporal dementia and its association with executive function. Neurobiology of Aging, 76, 62–70. 10.1016/j.neurobiolaging.2018.12.005, 30703627

[bib166] Zeng, X., & Luo, G. (2017). Progressive sampling-based Bayesian optimization for efficient and automatic machine learning model selection. Health Information Science and Systems, 5, 2. 10.1007/s13755-017-0023-z, 29038732 PMC5617811

[bib167] Zetterberg, H., van Swieten, J. C., Boxer, A. L., & Rohrer, J. D. (2019). Review: Fluid biomarkers for frontotemporal dementias. Neuropathology and Applied Neurobiology, 45(1), 81–87. 10.1111/nan.12530, 30422329

[bib168] Zhang, Y., Schuff, N., Du, A.-T., Rosen, H. J., Kramer, J. H., Gorno-Tempini, M. L., … Weiner, M. W. (2009). White matter damage in frontotemporal dementia and Alzheimer’s disease measured by diffusion MRI. Brain, 132, 2579–2592. 10.1093/brain/awp071, 19439421 PMC2732263

[bib169] Zhang, Y., Tartaglia, M. C., Schuff, N., Chiang, G. C., Ching, C., Rosen, H. J., … Weiner, M. W. (2013). MRI signatures of brain macrostructural atrophy and microstructural degradation in frontotemporal lobar degeneration subtypes. Journal of Alzheimer’s Disease, 33, 431–444. 10.3233/JAD-2012-121156, 22976075 PMC3738303

[bib170] Zheng, H., Yuan, J., & Chen, L. (2017). Short-term load forecasting using EMD-LSTM neural networks with a XGBoost algorithm for feature importance evaluation. Energies, 10(8), 1168. 10.3390/en10081168

[bib171] Zhou, J., Gennatas, E. D., Kramer, J. H., Miller, B. L., & Seeley, W. W. (2012). Predicting regional neurodegeneration from the healthy brain functional connectome. Neuron, 73, 1216–1227. 10.1016/j.neuron.2012.03.004, 22445348 PMC3361461

[bib172] Zhou, X., Sakaie, K. E., Debbins, J. P., Narayanan, S., Fox, R. J., & Lowe, M. J. (2018). Scan-rescan repeatability and cross-scanner comparability of DTI metrics in healthy subjects in the SPRINT-MS multicenter trial. Magnetic Resonance Imaging, 53, 105–111. 10.1016/j.mri.2018.07.011, 30048675 PMC6138530

[bib173] Zou, Q.-H., Zhu, C.-Z., Yang, Y., Zuo, X.-N., Long, X.-Y., Cao, Q.-J., … Zang, Y.-F. (2008). An improved approach to detection of amplitude of low-frequency fluctuation (ALFF) for resting-state fMRI: Fractional ALFF. Journal of Neuroscience Methods, 172, 137–141. 10.1016/j.jneumeth.2008.04.012, 18501969 PMC3902859

