# Supplementary material for: Multiclass characterization of frontotemporal dementia variants via multimodal brain network computational inference
Source: Netw Neurosci. 2023 Jan 1;7(1):322–50. doi: 10.1162/netn_a_00285 (PMC10270711; doi:10.1162/netn_a_00285)
Supplement: Supplementary file 1 [file netn-7-1-322-s001.pdf]

Gonzalez-Gomez, R., Ibanez, A. & Moguiliner, S. (2023). Supporting information for "Multiclass characterization of frontotemporal dementia variants via multimodal brain network computational inference." *Network Neuroscience*, 7(1), 322–350. https://doi.org/10.1162/netn_a_00285

Supplementary Material

1. **Supplementary data 1. Atrophy patterns**
2. **Supplementary data 2. Patient sample details**
3. **Supplementary data 3. Patient sample harmonization**
4. **Supplementary data 4. Models' performance**
5. **Supplementary data 5. Raw connectivity results details**

1. Supplementary data 1. Atrophy patterns

The SBM analysis was used with the subjects' T1 acquisitions to obtain cortical thickness values and characterize the atrophy in every group. All structural T1-weighted images were 3D scans, registered in the sagittal plane with an inversion recovery pulse sequence and the following parameters: TR = 2300 ms, TE = 3.0 ms, flip angle = 9.0 degree, voxel size = 1 mm^3, number of slices = 256. The preprocessing and surface-based morphometry analysis were implemented in CAT12 (http://www.neuro.uni-jena.de/cat), running in SPM12 on MATLAB R2018b. First, the images were segmented and normalized based on a surface and thickness estimation. Then, cortical thickness data was resampled and smoothed using a 12 mm kernel and merging both hemispheres. Second, we checked data quality, testing the sample homogeneity, and checking orthogonality. Third, the variants were compared with their respective HC subsamples through factorial *t*-contrasts. The multiple comparisons problem correction was performed with the threshold-free cluster enhancement (TFCE) method (Smith & Nichols, 2009) and the TFCE toolbox (http://www.neuro.uni-jena.de/tfce), which is an extension of SPM12. The cortical

thickness comparisons were corrected by the TFCE method with 5000 permutations and a statistical significance level of $p < 0.05$ (FWE-corrected).

We find the expected atrophy patterns for each FTD variant (**Supplementary Figure 1,** details in **Supplementary Table 1**). In bvFTD, cortical thickness decreased strongly in the frontal lobe and the anterior temporal region, as well as in parietal areas but with less affectation (Boxer et al., 2006; Seeley et al., 2009; Whitwell et al., 2009). The CBS showed damage mainly in the areas near the central sulcus, extending to the parietal, temporal, and frontal areas (Seeley et al., 2009; Whitwell et al., 2010). The nfvPPA and PSP groups presented atrophy in the frontal lobe, mainly in superior regions, including premotor and motor areas (Boxer et al., 2006; Lu et al., 2013; Seeley et al., 2009). This damage was lateralized to the right hemisphere in the case of PSP. In patients with svPPA, an anteroposterior gradient of temporal atrophy was detected, extensible to frontal areas like the insular and orbitofrontal cortex, especially in the left hemisphere (Lu et al., 2013; Seeley et al., 2009).

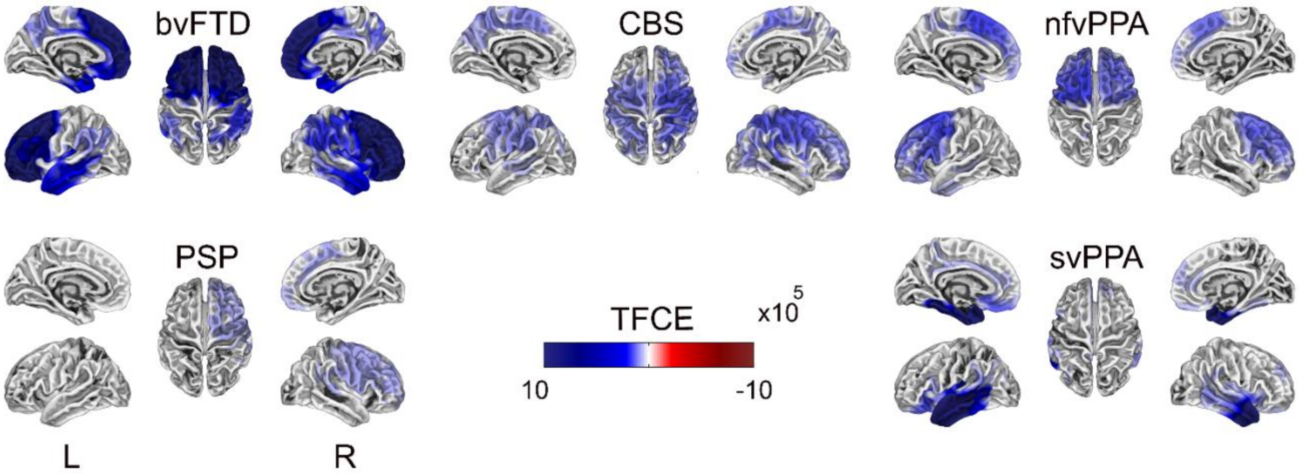

Supplementary Figure 1. Atrophy patterns for five frontotemporal dementia variants. Cortical thickness was calculated through surface-based morphometry TFCE, $p_{FEW} < 0.05$. Every variant was

compared to a sample of healthy controls matched in sex, age, and years of education. bvFTD: behavioral variant, CBS: cortico-basal syndrome, L: left, nfvPPA: nonfluent variant primary progressive aphasia, PSP: progressive supranuclear palsy, p_{FWE}: probability corrected by family-wise error, R: right, svPPA: semantic variant primary progressive aphasia. TFCE: threshold-free cluster enhancement.

Supplementary Table 1. Significate results on cortical thickness from the comparisons of five FTD variants with their respective HC group.

		**Coordinates**					
**Group**	**Region**	**x**	**y**	**z**	**K_E**	**TFCE**	**peak p_{FWE}**
bvFTD	Frontal Middle R	31	41	23	26720	1712411	<0.001
bvFTD	Cingulum Middle L	-12	23	32	26854	1236949	<0.001
CBS	Postcentral R	53	-20	51	21842	390635	<0.001
CBS	Precentral L	-33	-8	48	19799	297289	<0.001
CBS	Lingual L	-11	-67	-3	348	35736	0.007
nfvPPA	Precentral L	-49	0	48	19818	452448	<0.001
nfvPPA	Frontal Middle R	39	9	41	14992	360677	<0.001
nfvPPA	Occipital Inf L	-42	-76	-4	105	33579	0.006
nfvPPA	Occipital Sup L	-9	-103	5	93	33771	0.006
PSP	Frontal Mid R	38	3	34	14929	225572	<0.001
PSP	Frontal Inf Tri L	-38	27	4	7812	97746	0.001
PSP	Temporal Mid L	-51	-37	-7	1115	46623	0.005
PSP	Calcarine L	-4	-63	17	574	40089	0.006
PSP	Lingual L	-8	-49	46	140	39436	0.006
PSP	Lingual L	-7	-69	-2	1016	32111	0.009
svPPA	Temporal Pole Sup L	-49	12	-18	17965	3780106	<0.001
svPPA	Temporal Pole Mid R	39	19	-35	18559	1655185	<0.001

bvFTD: behavioral variant, CBS: cortico-basal syndrome, K_E: number of vertices, L: left, nfvPPA: nonfluent variant primary progressive aphasia, PSP: progressive supranuclear palsy, p_{FWE}: probability corrected by family-wise error, R: right, svPPA: semantic variant primary progressive aphasia. TFCE: threshold-free cluster enhancement.

2. Supplementary data 2. Patient sample details

Each binary comparison between groups, demographic, disease progression and cognitive variables is shown in **Supplementary Table 2**, accompanied by its respective statistical analysis to check for statistically significant differences. Additionally, **Supplementary Table 3** shows the statistical power for detecting medium effect sizes on all the group comparisons, with a minimum power of 0.80 as the recommended threshold (Cohen, 1988).

Supplementary Table 2. Demography, disease progression and cognition comparison among groups of five FTD variants and HC.

**Comparison**	**Age** (*p* [$CI_{0.95}$])	**Sex** (*p* [χ^2])	**Education** (p [$CI_{0.95}$])	**CDR** (*p* [$CI_{0.95}$])	**MMSE** (*p* [$CI_{0.95}$])
HC vs bvFTD	**0.038** [0,8]	**0.047** [3.76]	0.067 [0,4]	**<0.001** [-2,1]	**<0.001** [3,8]
HC vs CBS	0.760 [-6,5.5]	0.945 [< 0,001]	0.830 [-1,3]	**<0.001** [-1,-0.5]	**<0.001** [1.5,9]
HC vs nfvPPA	0.190 [-10,2]	1.000 [< 0,001]	0.830 [-1,4]	**<0.001** [-0.5,-0.5]	**<0.001** [1,7]
HC vs PSP	**0.050** [-7.5,1]	0.756 [0.09]	0.067 [0,4]	**<0.001** [-1,-0.5]	**<0.001** [1,6.5]
HC vs svPPA	0.300 [-2.5,7]	0.402 [0.70]	0.830 [-2,4]	**<0.001** [-1,-0.5]	**<0.001** [2.5,8]
bvFTD vs CBS	0.150 [-12,2]	0.089 [2.89]	0.830 [-4,2]	0.064 [0,1.5]	0.850 [-5,4.5]
bvFTD vs nfvPPA	**0.008** [-15,-1]	0.188 [1.73]	0.830 [-4,2.5]	**0.002** [0.5,1.5]	0.780 [-6,2]
bvFTD vs PSP	**0.008** [-12,-2]	0.252 [1.31]	0.850 [-2,2.5]	0.220 [0,1.5]	0.850 [-6,2.5]
bvFTD vs svPPA	0.360 [-7.5,4]	0.531 [0.39]	0.830 [-4,2]	0.940 [0,1.5]	0.780 [-4,4]
CBS vs nfvPPA	0.460 [-11,5.5]	0.913 [0.01]	0.90 [-2,4]	0.940 [0,0.5]	0.850 [-5.5,3]
CBS vs PSP	0.580 [-9.5,4]	0.692 [0.15]	0.850 [-1,4]	0.940 [-0.5,0.5]	0.850 [-5,3]
CBS vs svPPA	0.460 [-4,9.5]	0.680 [0.41]	0.900 [-2,2]	0.240 [-0.5,0.5]	0.620 [-4,5]
nfvPPA vs PSP	0.760 [-6,7]	0.980 [< 0,001]	0.850 [-2,4]	0.940 [-0.5,0]	0.850 [-3,3.5]
nfvPPA vs svPPA	**0.050** [-1,13]	0.646 [0.21]	0.900 [-4,3]	0.210 [-0.5,0]	0.300 [-2,5]
PSP vs svPPA	**0.025** [0,11]	0.805 [0.06]	0.830 [-4,2]	0.340 [-0.5,0.5]	0.450 [-2,5]

For the discrete variables, we used group's medians comparisons based on a 5000 resample permutations test. The results are shown as the 0.95 confidence interval for the difference between medians and their respective *p*-value. For the dichotomic variables, the equality of proportion between groups was analyzed with chi-square and its respective *p*-value. The *p* values were set to 0.05 and adjusted by the FDR method, in bold font the significant results. bvFTD: behavioral variant, CBS: corticobasal syndrome, HC: healthy controls, nfvPPA: nonfluent variant primary progressive aphasia, PSP: progressive supranuclear palsy, svPPA: semantic variant primary progressive aphasia.

Supplementary Table 3. Statistical power for detecting medium effect sizes on the calculated group comparisons.

Groups	HC_{sub1}	HC_{sub2}	bvFTD	CBS	nfvPPA	PSP	svPPA
bvFTD	0.85	-	-	-	-	-	-
CBS	-	0.83	0.82	-	-	-	-
nfvPPA	-	0.82	0.81	0.80	-	-	-
PSP	-	-	0.83	0.81	0.81	-	-
svPPA	0.83	0.84	0.82	0.81	0.80	0.81	-

bvFTD: behavioral variant, CBS: corticobasal syndrome, HC: healthy controls, nfvPPA: nonfluent variant primary progressive aphasia, PSP: progressive supranuclear palsy, svPPA: semantic variant primary progressive aphasia.

3. Supplementary data 3. Patient sample harmonization

To reduce possible biases in our samples, the patient groups were matched with two subsamples of HCs. **Supplementary Table 4** summarizes the statistical analyses of these comparisons, with two HC subgroups of n = 50. While **Supplementary Table 5** presents the same comparison for the subjects included in the DWI. In this case, the sample size of the HC subgroup was HC_{sub1}=43 and HC_{sub2}=41.

Supplementary Table 4. Demographic comparisons of five FTD variants with their respective HC group for data used in fMRI analysis.

	**Match subsample**	**Age** (p [$CI_{0.95}$])	**Sex** (p [$\chi 2$])	**Education** (p [$CI_{0.95}$])
bvFTD	HC_{sub1}	0.55 [-4, 3]	0.23 [1.47]	0.41 [-3, 0]
CBS	HC_{sub2}	0.72 [-6, 3.5]	0.78 [0.08]	0.77 [-2, 2]
nfvPPA	HC_{sub2}	0.44 [-6,3]	0.81 [0.06]	0.88 [-2, 2.5]
PSP	HC_{sub2}	0.64 [-4, 2]	0.58 [0.31]	0.29 [0,3]
svPPA	HC_{sub1}	0.55 [-5, 2]	1.00 [< 0,001]	0.71 [-2, 2]

For the discrete variables we used group median comparisons based on a 5000 permutations test, to deal with tied values (Wilcox, 2017). The results are shown as the 0.95 confidence interval for the difference between medians and their respective *p*-value. For the dichotomic variables, the equality of proportion between groups was analyzed with chi-square and its respective *p*-value. bvFTD: behavioral variant, CBS: corticobasal syndrome, HC: healthy controls, nfvPPA: nonfluent variant primary progressive aphasia, PSP: progressive supranuclear palsy, svPPA: semantic variant primary progressive aphasia.

Supplementary Table 5. Demographic comparisons of five FTD variants with their respective HC group for data used in DWI analysis

	**Match subsample**	**Age** (p [$CI_{0.95}$])	**Sex** (p [$\chi 2$])	**Education** (p [$CI_{0.95}$])
bvFTD	HC_{sub1}	0.67 [-5, 3]	0.30 [1.09]	0.36 [-2.5, 0]
CBS	HC_{sub2}	0.64 [-6, 3]	0.89 [0.02]	0.86 [-1, 2]
fvPPA	HC_{sub2}	0.41 [-6,3]	0.80 [0.06]	0.97 [-2, 2]
PSP	HC_{sub2}	0.72 [-4, 2]	0.68 [0.17]	0.51 [0,3]
svPPA	HC_{sub1}	0.62 [-5, 3]	1.0 [< 0,001]	0.72 [-2, 2]

For the discrete variables, we used group median comparisons based on a 5000 permutations test, to deal with tied values (Wilcox, 2017). The results are shown as the 0.95 confidence interval for the difference between medians and their respective *p*-value. For the dichotomic variables, the equality of proportion between groups was analyzed with chi-square and its respective *p*-value. bvFTD: behavioral variant, CBS: corticobasal syndrome, HC: healthy controls, nfvPPA: nonfluent variant primary progressive aphasia, PSP: progressive supranuclear palsy, svPPA: semantic variant primary progressive aphasia.

4. Supplementary data 4. Models' performance

Performance details for all models are presented in **Supplementary Table 6** and **Supplementary Table 7** shows the AUC variability by class through the cross validation. Additionally, we computed all the classifiers with data from scanner 1 (n

= 285), see **Supplementary Table 8,** to avoid any potential biases due to different acquisition parameters**.** The micro-averages AUC were not statistically different for any classifier using two or one scanner (**Supplementary Table 9**). Nonparametric tests were implemented to assess statistically significant differences between the ROC curves (Venkatraman, 2000). The variability of the feature importance list was evaluated across nested k-folds to confirm whether the confidence interval of each feature was ranked in the same way as the feature mean. This approach was employed for all modalities, including functional (**Supplementary figure 2**), structural (**Supplementary figure 3**), and combined (**Supplementary figure 4**). Result confirmed the stability of the selected features.

Supplementary Table 6. Performance metrics of the ML models. Mean and confidence intervals for sensitivity, specificity, accuracy, F1, and AUC for the fourteen ML models employed.

Models	Accuracy	Sensitivity	Specificity	F1	AUC*	Average
F. Raw C	0.90 [0.89 0.92]	0.89 [0.86 0.91]	0.91 [0.88 0.94]	0.90 [0.88 0.91]	0.92 [0.89 0.93]	0.90 [0.86 0.94]
F. Raw MF	0.93 [0.92 0.94]	0.93 [0.92 0.95]	0.92 [0.91 0.95]	0.89 [0.88 0.90]	0.95 [0.94 0.97]	0.92 [0.88 0.97]
F. ROI-to-ROI C	0.76 [0.75 0.77]	0.74 [0.69 0.78]	0.77 [0.73 0.78]	0.73 [0.72 0.75]	0.75 [0.72 0.77]	0.75 [0.69 0.78]
F. ROI-to-ROI MF	0.80 [0.79 0.82]	0.82 [0.81 0.83]	0.79 [0.75 0.82]	0.79 [0.76 0.81]	0.80 [0.78 0.83]	0.80 [0.75 0.83]
F. Graph C	0.62 [0.60 0.63]	0.65 [0.63 0.67]	0.61 [0.60 0.63]	0.61 [0.59 0.63]	0.63 [0.61 0.65]	0.62 [0.59 0.67]
F. Graph MF	0.87 [0.86 0.98]	0.88 [0.85 0.89]	0.86 [0.84 0.93]	0.86 [0.84 0.88]	0.89 [0.86 0.91]	0.87 [0.84 0.98]
S. Raw C	0.84 [0.82 0.85]	0.84 [0.82 0.85]	0.83 [0.81 0.85]	0.83 [0.81 0.85]	0.84 [0.83 0.87]	0.84 [0.81 0.87]
S. Raw MF	0.84 [0.83 0.86]	0.83 [0.82 0.86]	0.85 [0.80 0.88]	0.82 [0.80 0.84]	0.85 [0.81 0.87]	0.84 [0.80 0.88]
S. ROI-to-ROI C	0.72 [0.71 0.74]	0.72 [0.71 0.75]	0.73 [0.71 0.75]	0.69 [0.68 0.72]	0.70 [0.69 0.73]	0.72 [0.68 0.75]
S. ROI-to-ROI MF	0.80 [0.77 0.82]	0.81 [0.79 0.82]	0.79 [0.77 0.86]	0.79 [0.77 0.80]	0.80 [0.78 0.83]	0.80 [0.77 0.86]
S. Graph C	0.73	0.72	0.74	0.72	0.73	0.73

	[0.71 0.74]	[0.71 0.74]	[0.73 0.78]	[0.71 0.73]	[0.72 0.76]	[0.71 0.76]
S. Graph MF	0.81 [0.80 0.82]	0.81 [0.79 0.83]	0.81 [0.80 0.84]	0.81 [0.79 0.83]	0.83 [0.81 0.85]	0.81 [0.79 0.85]
Multimodal C	0.79 [0.77 0.82]	0.79 [0.77 0.82]	0.79 [0.77 0.84]	0.79 [0.77 0.82]	0.80 [0.78 0.83]	0.79 [0.77 0.84]
Multimodal MF	0.89 [0.86 0.90]	0.90 [0.89 0.92]	0.88 [0.86 0.93]	0.88 [0.86 0.91]	0.89 [0.85 0.92]	0.89 [0.85 0.93]

Statistics are presented as the mean [confidence intervals]. F: functional, MF: multifeature, C: Connectivity, S: structural, *micro-average AUC.

Supplementary Table 7. AUC's performance by class on ML models.

Models	HC	bvFTD	CBS	nfvPPA	PSP	svPPA
F. Raw C	0.94 [0.92 0.95]	0.72 [0.70 0.74]	0.97 [0.95 0.98]	0.76 [0.73 0.78]	0.95 [0.92 0.97]	0.97 [0.96 0.98]
F. Raw MF	0.99 [0.98 0.99]	0.89 [0.87 0.91]	0.94 [0.92 0.96]	0.89 [0.88 0.91]	0.88 [0.87 0.89]	0.99 [0.98 0.99]
F. ROI-to-ROI C	0.82 [0.80 0.84]	0.90 [0.89 0.92]	0.71 [0.70 0.73]	0.54 [0.53 0.55]	0.73 [0.71 0.75]	0.79 [0.76 0.82]
F. ROI-to-ROI MF	0.97 [0.96 0.98]	0.80 [0.78 0.82]	0.77 [0.75 0.79]	0.72 [0.71 0.73]	0.81 [0.79 0.82]	0.70 [0.69 0.72]
F. Graph C	0.63 [0.62 0.65]	0.80 [0.78 0.82]	0.58 [0.57 0.60]	0.55 [0.52 0.57]	0.66 [0.64 0.68]	0.49 [0.48 0.50]
F. Graph MF	0.98 [0.97 0.99]	0.93 [0.91 0.95]	0.82 [0.81 0.83]	0.85 [0.82 0.87]	0.79 [0.77 0.80]	0.85 [0.82 0.87]
S. Raw C	0.90 [0.88 0.92]	0.90 [0.88 0.92]	0.95 [0.92 0.97]	0.64 [0.63 0.65]	0.78 [0.76 0.80]	0.94 [0.92 0.96]
S. Raw MF	0.97 [0.95 0.98]	0.87 [0.86 0.88]	0.76 [0.74 0.78]	0.74 [0.72 0.76]	0.74 [0.73 0.76]	1.00 [0.99 1.00]
S. ROI-to-ROI C	0.81 [0.79 0.83]	0.59 [0.57 0.61]	0.74 [0.72 0.76]	0.63 [0.61 0.65]	0.59 [0.57 0.61]	0.80 [0.78 0.82]
S. ROI-to-ROI MF	0.93 [0.92 0.94]	0.88 [0.86 0.89]	0.80 [0.78 0.82]	0.55 [0.52 0.57]	0.79 [0.78 0.81]	0.81 [0.79 0.83]
S. Graph C	0.88 [0.85 0.89]	0.70 [0.68 0.72]	0.64 [0.63 0.65]	0.64 [0.61 0.66]	0.69 [0.67 0.71]	0.95 [0.92 0.97]
S. Graph MF	0.99 [0.98 0.99]	0.89 [0.88 0.92]	0.73 [0.71 0.75]	0.70 [0.69 0.72]	0.71 [0.70 0.72]	0.86 [0.84 0.87]
Multimodal C	0.94 [0.92 0.96]	0.82 [0.81 0.84]	0.76 [0.74 0.78]	0.62 [0.61 0.63]	0.80 [0.78 0.82]	0.81 [0.79 0.83]
Multimodal MF	0.97 [0.94 0.98]	0.93 [0.91 0.95]	0.95 [0.94 0.96]	0.85 [0.82 0.87]	0.80 [0.79 0.81]	0.86 [0.84 0.88]

Statistics are presented as the mean [confidence intervals]. bvFTD: behavioral variant, CBS: corticobasal syndrome, C: Connectivity, F: functional, HC: healthy controls, nfvPPA: nonfluent variant primary progressive aphasia, MF: multifeature, PSP: progressive supranuclear palsy, S: structural, svPPA: semantic variant primary progressive aphasia.

Supplementary Table 8. Performance metrics of the ML models with data from a single scanner. Mean and confidence intervals for sensitivity, specificity, accuracy, F1, and AUC for the fourteen ML models employed.

Models	Accuracy	Sensitivity	Specificity	F1	AUC*	Average
F. Raw C	0.91 [0.88 0.93]	0.88 [0.87 0.92]	0.93 [0.89 0.95]	0.92 [0.89 0.94]	0.93 [0.88 0.94]	0.91 [0.86 0.95]
F. Raw MF	0.93 [0.93 0.94]	0.93 [0.92 0.94]	0.93 [0.92 0.95]	0.90 [0.89 0.92]	0.94 [0.92 0.98]	0.93 [0.87 0.97]
F. ROI-to-ROI C	0.77 [0.76 0.78]	0.75 [0.72 0.76]	0.78 [0.74 0.80]	0.72 [0.71 0.74]	0.76 [0.72 0.79]	0.74 [0.69 0.78]
F. ROI-to-ROI MF	0.79 [0.78 0.82]	0.84 [0.82 0.85]	0.75 [0.73 0.79]	0.78 [0.76 0.82]	0.81 [0.77 0.83]	0.81 [0.75 0.85]
F. Graph C	0.63 [0.61 0.64]	0.67 [0.64 0.69]	0.62 [0.60 0.64]	0.61 [0.58 0.65]	0.62 [0.60 0.66]	0.63 [0.59 0.67]
F. Graph MF	0.86 [0.83 0.89]	0.90 [0.86 0.92]	0.88 [0.86 0.93]	0.85 [0.84 0.87]	0.88 [0.86 0.92]	0.87 [0.84 0.89]
S. Raw C	0.82 [0.83 0.84]	0.85 [0.83 0.87]	0.84 [0.80 0.85]	0.82 [0.80 0.85]	0.85 [0.83 0.88]	0.85 [0.82 0.86]
S. Raw MF	0.85 [0.84 0.87]	0.84 [0.83 0.87]	0.86 [0.82 0.87]	0.81 [0.79 0.84]	0.86 [0.82 0.91]	0.85 [0.80 0.88]
S. ROI-to-ROI C	0.72 [0.71 0.73]	0.73 [0.72 0.76]	0.74 [0.72 0.75]	0.70 [0.68 0.74]	0.72 [0.69 0.74]	0.71 [0.68 0.75]
S. ROI-to-ROI MF	0.81 [0.80 0.85]	0.84 [0.81 0.86]	0.80 [0.77 0.83]	0.78 [0.76 0.80]	0.81 [0.78 0.84]	0.82 [0.78 0.87]
S. Graph C	0.74 [0.73 0.75]	0.73 [0.72 0.74]	0.75 [0.73 0.77]	0.72 [0.71 0.74]	0.74 [0.72 0.76]	0.74 [0.72 0.76]
S. Graph MF	0.82 [0.81 0.84]	0.81 [0.78 0.84]	0.84 [0.82 0.86]	0.80 [0.78 0.83]	0.82 [0.81 0.84]	0.81 [0.78 0.86]
Multimodal C	0.78 [0.76 0.84]	0.79 [0.76 0.81]	0.78 [0.77 0.83]	0.81 [0.77 0.84]	0.81 [0.78 0.84]	0.80 [0.78 0.84]
Multimodal MF	0.90 [0.87 0.91]	0.91 [0.88 0.93]	0.89 [0.87 0.93]	0.87 [0.86 0.91]	0.88 [0.85 0.92]	0.88 [0.85 0.92]

Statistics are presented as the mean [confidence intervals]. F: functional, MF: multifeature, C: Connectivity, S: structural, *micro-average AUC.

Supplementary Table 9. Results of nonparametric tests to assess statistically differences between the ROC curves between two or a single scanner**.**

Models	Statistic	p value

F. Raw	1.56	0.18
F. Raw MF	1.54	0.19
F. ROI-to-ROI C	1.69	0.14
F. ROI-to-ROI MF	1.57	0.16
F. Graph C	1.44	0.22
F. Graph MF	1.39	0.24
S. Raw C	1.55	0.17
S. Raw MF	1.68	0.15
S. ROI-to-ROI C	1.46	0.21
S. ROI-to-ROI MF	1.33	0.25
S. Graph C	1.48	0.20
S. Graph MF	1.54	0.19
Multimodal C	1.71	0.13
Multimodal MF	1.73	0.12

F: functional, MF: multifeature, C: Connectivity, S: structural.

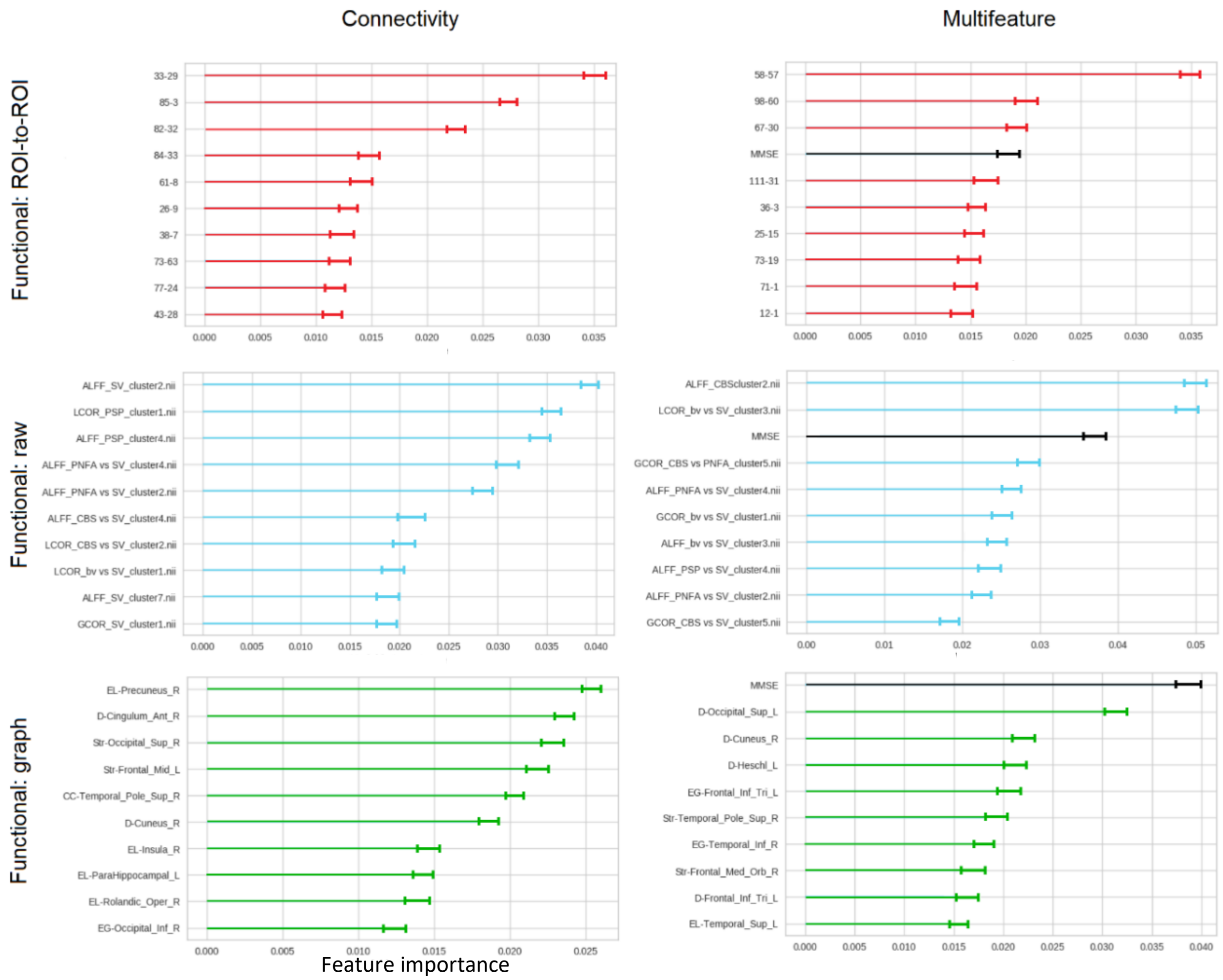

Supplementary figure 2. Feature variability analysis of functional models indicating confidence interval ranking under feature stabilization. ALFF: amplitude of low-frequency fluctuations, Ant: anterior, AUC: area under the ROC curve, bvFTD: behavioral variant, CBS: corticobasal syndrome, CC: clustering coefficient, D: degree, GCOR: global correlation, GL: global efficiency, HC: healthy controls, Inf: inferior, L: left, LCOR: local correlation, LE: local efficiency, nfvPPA: progressive nonfluent aphasia, Mid: middle, MMSE: Mini-Mental State Examination, oper: operculum, PSP: progressive supranuclear palsy, R: right, Sup: superior, svPPA: semantic variant of FTD, Str: strength, 1: Precentral L, 3: Frontal Sup L, 7: Frontal Mid L, 8: Frontal Mid R, 9: Frontal Mid Orb L, 12: Frontal Inf Oper R, 19: Supp Motor Area L, 20: Supp Motor Area R, 21: Olfactory L, 24: Frontal Sup Med R, 25: Frontal Med Orb L, 26: Frontal Med Orb R, 29: Insula L, 30: Insula R, 31: Cingulum Ant L, 32: Cingulum Ant R, 33: Cingulum Mid L, 36: Cingulum Post R, 38: Hippocampus R, 39: ParaHippocampal L, 43: Calcarine L, 57: Postcentral L, 58: Postcentral R, 60: Parietal Sup R, 61: Parietal Inf L, 63: SupraMarginal L, 67: Precuneus L, 71: Caudate L, 73: Putamen L, 77: Thalamus L, 82: Temporal Sup R, 84: Temporal Pole Sup R, 85: Temporal Mid L, 98: Cerebelum 4 5 R, 111: Vermis 4 5.

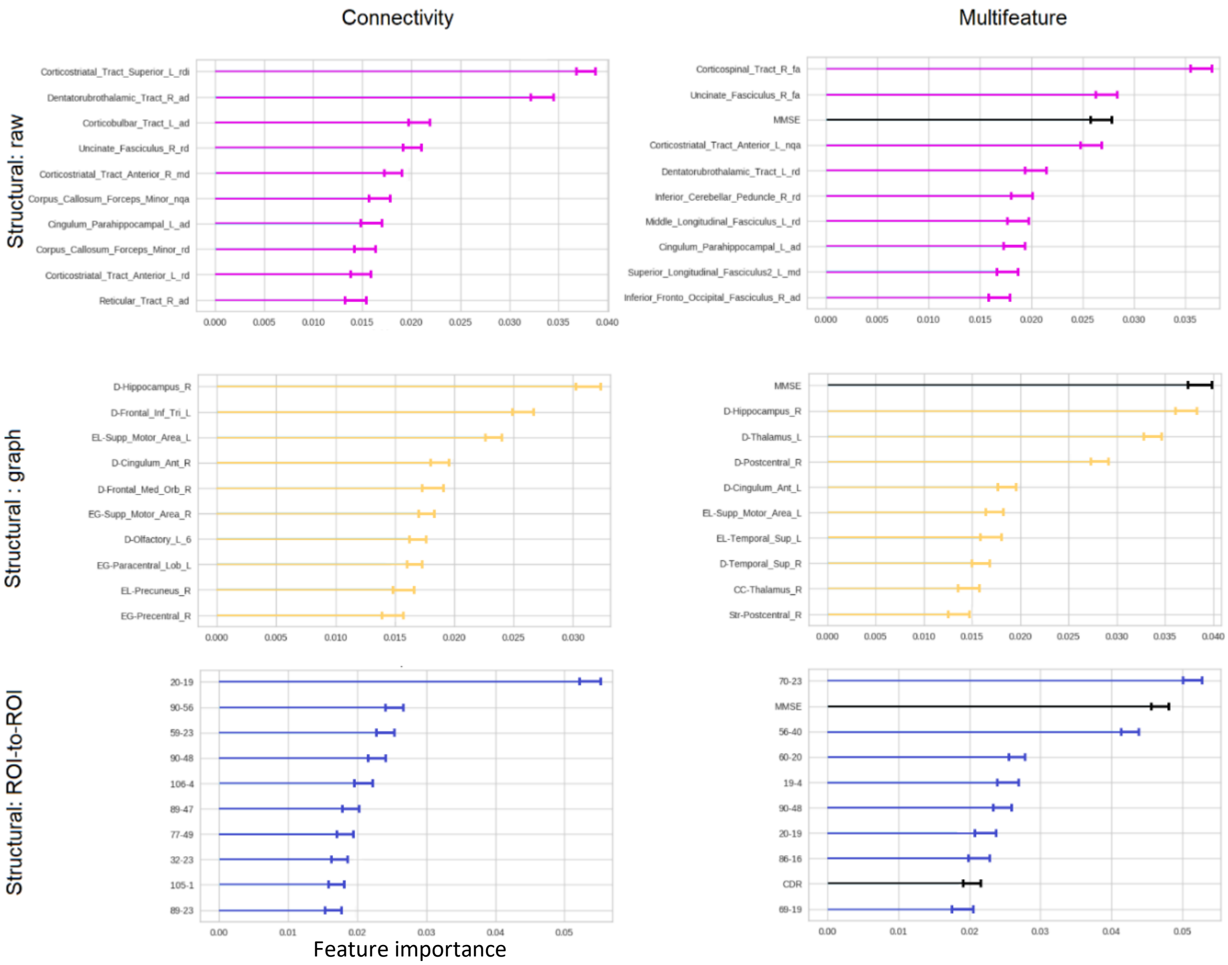

Supplementary figure 3. Feature variability analysis of structural models indicating confidence interval ranking under feature stabilization. Ant: anterior, AUC: area under the ROC curve, bvFTD: behavioral variant, CBS: corticobasal syndrome, CC: clustering coefficient, Cereb: cerebellum, Cing: cingulum, D: degree, FA: fractional anisotropy, , Fr: frontal, GL: global efficiency, HC: healthy controls, Hipp: hippocampus, Inf: inferior, L: left, LE: local efficiency, Long: longitudinal, MD: mean diffusivity, Med: medial, NQA: normalized quantitative anisotropy, nfvPPA: progressive nonfluent aphasia, MMSE: Mini-Mental State Examination, Orb: orbital, PSP: progressive supranuclear palsy, R: right, RD: radial diffusivity, Sup: superior, Supp: supplementary, svPPA: semantic variant of FTD, Str: strength, Temp: temporal, Tri: pars triangularis, 1: Precentral L, 4: Frontal Sup R, 16: Frontal Inf Orb R, 19: Supp Motor Area L, 20: Supp Motor Area R, 23: Frontal Sup Med L, 32: Cingulum Ant R, 40: ParaHippocampal R, 47: Lingual L, 48: Lingual R, 49: Occipital Sup L, 56: Fusiform R, 60: Parietal Sup R, 69: Paracentral Lob L, 70: Paracentral Lob R, 77: Thalamus L, 86: Temporal Mid R, 89: Temporal Inf L, 90: Temporal Inf R, 105: Cerebelum 9 L.

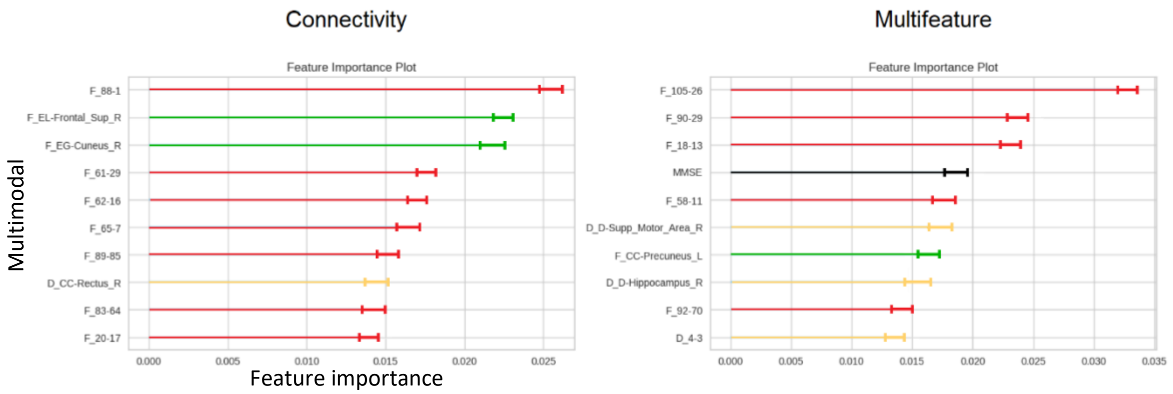

Supplementary figure 4. Feature variability analysis of multimodal models indicating confidence interval ranking under feature stabilization. bvFTD: behavioral variant, CBS: corticobasal syndrome, CC: clustering coefficient, D: degree, F: functional, GL: global efficiency, HC: healthy controls, L: left, LE: local efficiency, nfvPPA: progressive nonfluent aphasia, Med: medial, Mid: middle, MMSE: Mini-Mental State Examination, PSP: progressive supranuclear palsy, R: right, Rolan: rolandic, S: structural, Sup: superior, sup: supplementary, svPPA: semantic variant of FTD, 1: Precentral L, 3: Frontal Sup L, 4: Frontal Sup R, 7: Frontal Mid L, 13: Frontal Inf Tri L, 17: Rolandic Oper L, 18: Rolandic Oper R, 20: Supp Motor Area R, 26: Frontal Med Orb R, 29: Insula L, 58: Postcentral R, 61: Parietal Inf L, 62: Parietal Inf R, 64: SupraMarginal R, 70: Paracentral Lob R, 83: Temporal Pole Sup L, 85: Temporal Mid L, 89: Temporal Inf L, 90: Temporal Inf R, 92: Cerebelum Crus1 R, 105: Cerebelum 9 L.

5. Supplementary data 5. Raw connectivity results details

The statistical results of the raw connectivity analysis are shown in **Supplementary Table 10** and **Supplementary Figure 5** for functional and structural analysis, respectively.

Supplementary Table 10. Significant clusters of raw connectivity from the comparisons of five FTD variants with their respective HC groups.

			Coordinates					
**Group**	**Cluster**	**Region**	**x**	**y**	**z**	**size**	**TFCE**	**peak p_{FWE}**
			**Global correlation**					
bvFTD	1	Frontal Pole L	4	48	18	11274	-2498.8	0.000
bvFTD	2	Precuneus	6	-66	54	4270	1600.9	0.000
bvFTD	3	Temporal Pole R	58	12	-28	2612	-1242.0	0.001

bvFTD	4	Temporal Inferior R	54	-42	-22	2316	1101.7	0.003
bvFTD	5	Caudate L	-12	12	6	206	-963.6	0.011
bvFTD	6	Temporal Inferior L	-54	-54	-28	1165	960.3	0.011
bvFTD	7	Parietal Inferior L	36	-28	30	64	890.4	0.024
bvFTD	8	Parietal Inferior R	-30	-54	42	72	838.0	0.035
bvFTD	9	Occipital Lateral L	-26	-76	26	67	810.4	0.041
bvFTD	10	Vermis 4_5	0	-54	-22	7	787.6	0.050
bvFTD	11	Occipital Pole L	-18	-96	-18	36	786.3	0.050
CBS	1	Frontal Pole L	-20	44	12	747	1013.8	0.009
CBS	2	Caudate L	-18	-12	30	390	986.0	0.009
CBS	3	Frontal Middle R	36	24	30	158	891.8	0.015
CBS	4	Frontal Middle R	22	24	18	95	827.2	0.035
CBS	5	Cerebellum 9 L	-6	-60	-48	42	-786.3	0.044
CBS	6	Cerebellum 9 R	10	-60	-46	68	-783.2	0.046
CBS	7	Cerebellum 6 R	24	-54	-22	7	-776.5	0.046
CBS	8	Cerebellum 8 L	-12	-66	-58	7	-769.7	0.049
nfvPPA	1	Insula L	-42	-12	6	814	-1211.2	0.002
nfvPPA	2	Temporal Pole L	-54	-6	-12	900	1028.8	0.009
PSP	1	Insula L	-42	-12	6	1936	-1515.5	0.000
PSP	2	Postcentral L	-62	-12	30	555	-923.4	0.011
PSP	3	Temporal Pole L	-30	2	-24	102	-834.2	0.024
PSP	4	Caudate L	-12	18	6	172	-819.2	0.027
PSP	5	Insula L	-36	18	-4	97	-789.5	0.029
PSP	6	Temporal Pole R	-18	-46	54	2	-741.7	0.049
svPPA	1	Temporal Pole L	36	20	-36	6788	-4201.6	0.000
svPPA	2	Cerebellum Crus1 L	-48	8	-34	4987	-3096.1	0.000
svPPA	3	Cerebellum Crus2 L	-12	-92	-30	400	827.4	0.031
svPPA	4	Cerebellum Crus1 R	30	-84	-30	63	776.6	0.037
svPPA	5	Precuneus	10	-66	42	32	763.2	0.044
bvFTD vs. CBS	1	Frontal Pole L	-18	42	6	15620	-2454.7	0.000
bvFTD vs. CBS	2	Cerebellum 6 R	-18	-58	-22	2439	1289.4	0.001
bvFTD vs. CBS	3	Frontal Middle R	54	24	20	692	-976.3	0.013
bvFTD vs. CBS	4	Cerebellum 8 R	-6	-48	-60	576	954.3	0.015
bvFTD vs. CBS	5	Frontal Pole R	48	42	-12	98	-760.9	0.048
bvFTD vs. nfvPPA	1	Paracingulate L	0	44	12	278	-926.2	0.022
bvFTD vs. PSP	2	Frontal Pole L	-6	42	2	3535	-1383.1	0.000
bvFTD vs. PSP	3	Occipital Lateral L	-14	-48	54	321	857.1	0.029
bvFTD vs. PSP	4	Precuneus L	-26	-52	6	254	842.0	0.032
bvFTD vs. PSP	5	Cingulum Anterior L	-14	-36	36	69	776.9	0.043
bvFTD vs. svPPA	1	Frontal Pole L	-32	56	6	1346	-1135.3	0.009
bvFTD vs. svPPA	2	Temporal Pole R	24	12	-30	684	1070.5	0.010
bvFTD vs. svPPA	3	Fusiform R	-24	-4	-42	359	1038.5	0.011
bvFTD vs. svPPA	4	Temporal Pole R	-18	12	-36	74	858.0	0.028

bvFTD vs. svPPA	5	Temporal Pole R	-44	12	-36	75	846.3	0.029
CBS vs. nfvPPA	1	Frontal Pole L	-30	48	0	1162	1243.0	0.002
CBS vs. nfvPPA	2	Thalamus L	-8	-10	24	724	967.5	0.014
CBS vs. nfvPPA	3	Vermis 8	0	-70	-40	90	-910.4	0.023
CBS vs. nfvPPA	4	Frontal Inferior L	-48	18	-4	385	908.2	0.023
CBS vs. nfvPPA	5	Frontal Middle L	-42	14	44	93	842.2	0.038
CBS vs. PSP	1	Insula L	-8	26	6	2620	1203.3	0.004
CBS vs. PSP	2	Thalamus L	-30	-30	-12	2328	1045.4	0.011
CBS vs. PSP	3	Insula L	-36	14	-6	116	847.2	0.039
CBS vs. PSP	4	Frontal Pole L	-20	36	30	101	832.9	0.039
CBS vs. svPPA	1	Temporal Pole L	42	20	-30	1682	1629.1	0.000
CBS vs. svPPA	2	Temporal Pole R	-44	12	-34	889	1379.8	0.000
CBS vs. svPPA	3	Cerebellum 6 L	4	-66	-42	671	-998.5	0.018
CBS vs. svPPA	4	Fusiform L	-30	-28	-12	220	901.4	0.030
CBS vs. svPPA	5	Cingulum Anterior L	-6	24	-6	206	879.9	0.030
nfvPPA vs. PSP	1	Postcentral L	-14	-48	54	186	1170.3	0.028
nfvPPA vs. svPPA	1	Temporal Pole R	28	-6	-36	3243	1232.8	0.002
PSP vs. svPPA	1	Temporal Pole R	36	12	-50	3139	1587.8	0.001
PSP vs. svPPA	2	Temporal Pole L	-48	12	-34	948	1177.1	0.006
PSP vs. svPPA	3	Poscentral L	-14	-24	36	695	-1063.8	0.012
PSP vs. svPPA	4	Precuneus L	-30	-48	6	645	-1043.8	0.014
PSP vs. svPPA	5	Parietal Operculum L	-42	-10	6	917	1001.5	0.017
PSP vs. svPPA	6	Frontal Medial R	12	30	-6	410	892.7	0.031
**Local correlation**								
bvFTD	1	Frontal Pole	-20	20	12	27876	4893.6	0.000
bvFTD	2	Cerebellum 8 R	30	-84	-34	7589	-1604.0	0.000
bvFTD	3	Temporal Pole R	54	20	-10	673	-1456.8	0.000
bvFTD	4	Putamen R	24	2	-8	525	-1060.6	0.007
bvFTD	5	Temporal Pole L	-48	24	-18	123	-922.9	0.016
bvFTD	6	Calcarine L	-20	-66	8	152	-840.3	0.033
bvFTD	7	Putamen L	-22	2	-4	88	-799.0	0.042
bvFTD	8	Precentral R	64	0	18	8	-772.7	0.046
bvFTD	9	Postcentral R	66	-12	12	29	-762.8	0.048
bvFTD	10	Precuneus	-12	-66	18	1	-760.6	0.049
bvFTD	11	Precuneus	-12	-66	22	1	-758.9	0.050
CBS	1	Thalamus R	42	-40	-10	14640	3894.8	0.000
CBS	2	Frontal Pole L	-22	54	10	2770	1487.1	0.000
CBS	3	Cerebellum Crus2 R	34	-84	-36	1870	-1084.8	0.005
CBS	4	Postcentral L	-42	-44	58	1016	-983.7	0.010
CBS	5	Frontal Pole R	22	50	2	143	798.3	0.034
CBS	6	Postcentral L	-62	-24	38	15	-774.5	0.043
CBS	7	Paracingulate L	-12	24	38	32	768.2	0.046
CBS	8	Cerebellum Crus2 L	-12	-76	-40	6	-762.3	0.049

nfvPPA	1	Thalamus L	-20	6	24	6126	1811.6	0.001
nfvPPA	2	Cerebellum 8 R	24	-52	-42	298	-912.8	0.020
nfvPPA	3	Cerebellum Crus2 R	32	-78	-30	506	-898.5	0.021
PSP	1	Frontal Pole R	52	-50	-12	16939	1927.3	0.000
PSP	2	Cerebellum Crus2 L	18	-88	-42	3688	-1742.3	0.000
PSP	3	Thalamus L	-10	-4	6	44	-836.8	0.033
PSP	4	Frontal Pole L	-30	42	-12	98	835.6	0.033
PSP	5	Frontal Pole L	-20	54	2	211	833.2	0.033
PSP	6	Putamen L	-24	0	-4	220	-785.7	0.043
svPPA	1	Frontal Pole L	-26	-36	6	11378	2078.3	0.000
svPPA	2	Putamen L	-20	2	-8	2052	-1484.2	0.001
svPPA	3	Temporal Pole R	52	22	-12	309	-1055.2	0.009
svPPA	4	Cerebellum 8 L	-26	-42	-58	443	-1003.7	0.013
svPPA	5	Cerebellum 8 R	18	-60	-60	79	-829.3	0.038
svPPA	6	Cerebellum 8 R	32	-48	-58	19	-803.6	0.042
svPPA	7	Cerebellum 8 R	36	-66	-54	13	-798.2	0.043
svPPA	8	Cerebellum 8 R	12	-66	-58	17	-795.3	0.043
svPPA	9	Cerebellum 8 R	22	-66	-54	3	-786.1	0.049
bvFTD vs. CBS	1	Frontal Superior R	0	26	50	89	899.6	0.035
bvFTD vs. CBS	2	Caudate L	-18	26	-2	89	873.6	0.042
bvFTD vs. nfvPPA	1	Frontal Superior R	4	32	36	1779	1301.7	0.000
bvFTD vs. PSP	1	Frontal Superior R	4	26	36	3972	1572.4	0.000
bvFTD vs. svPPA	1	Frontal Orbital L	-30	26	-4	7515	1761.2	0.000
bvFTD vs. svPPA	2	Precentral L	-36	-10	54	525	-966.8	0.018
bvFTD vs. svPPA	3	Cerebellum Crus2 L	-24	-88	-34	140	-885.6	0.031
bvFTD vs. svPPA	4	Angular L	-48	-66	26	263	-869.9	0.033
CBS vs. nfvPPA	1	Postcentral L	-36	-36	62	1433	-1375.0	0.000
CBS vs. svPPA	2	Thalamus R	18	-30	24	1752	1160.4	0.000
PSP vs. svPPA	1	Insula L	-26	6	12	134	945.0	0.000
PSP vs. svPPA	2	Insula R	40	2	12	104	849.7	0.000
**Amplitude of low-frequency fluctuations**								
bvFTD	1	Frontal Pole L	-6	12	20	21191	5840.5	0.000
bvFTD	2	Precuneus	0	-42	24	5945	-1845.7	0.000
bvFTD	3	Putamen R	24	6	-6	771	-1178.7	0.000
bvFTD	4	Supramarginal R	64	-36	36	1312	-1062.4	0.002
bvFTD	5	Putamen L	-24	0	2	231	-1023.2	0.002
bvFTD	6	Frontal Middle L	-30	0	60	150	-954.4	0.007
bvFTD	7	Frontal Inferior R	46	26	12	311	831.8	0.013
bvFTD	8	Frontal Pole L	-38	12	12	140	818.9	0.015
bvFTD	9	Temporal Pole R	56	18	-10	261	-797.8	0.023
bvFTD	10	Frontal Middle R	34	6	48	288	-781.8	0.025
bvFTD	11	Angular L	-60	-54	20	143	-749.3	0.033
bvFTD	12	Supramarginal L	-66	-34	30	47	-744.3	0.033

bvFTD	13	Putamen L	-26	-16	6	28	-719.7	0.042
bvFTD	14	Precentral L	-26	-12	54	45	-719.4	0.042
bvFTD	15	Thalamus L	0	-6	8	19	-715.5	0.042
bvFTD	16	Postcentral R	22	-40	54	55	-715.3	0.042
bvFTD	17	Postcentral R	46	-12	36	19	-707.5	0.043
bvFTD	18	Frontal Middle R	48	18	42	50	-696.5	0.044
bvFTD	19	Frontal Pole R	24	56	32	21	690.1	0.047
bvFTD	20	Supramarginal L	-56	-46	42	27	-686.9	0.048
CBS	1	Temporal fusiform R	34	-36	12	1796	1174.8	0.000
CBS	2	Parietal Inferior L	-44	-24	26	2105	1123.0	0.000
CBS	3	Frontal Pole L	-24	54	12	450	986.7	0.003
CBS	4	Frontal Orbital R	22	20	-30	56	-746.2	0.028
CBS	5	Putamen R	24	2	0	191	-743.6	0.028
CBS	6	Amygdala R	16	0	-22	6	-668.1	0.048
nfvPPA	1	Frontal Pole L	-18	26	12	6787	1545.9	0.001
nfvPPA	2	Caudate R	18	6	32	638	848.7	0.011
nfvPPA	3	Brain Stem	-2	-6	-24	42	-682.5	0.039
nfvPPA	4	Hippocampus R	36	-36	-6	22	670.8	0.044
PSP	1	Temporal Pole L	-6	-6	-6	10171	1648.3	0.000
PSP	2	Putamen R	24	0	6	1914	-1271.7	0.001
PSP	3	Frontal Superior L	4	24	30	1992	-1190.6	0.001
PSP	4	Frontal Pole L	-24	54	8	505	879.9	0.011
PSP	5	Frontal Middle R	30	18	44	668	-875.7	0.011
PSP	6	Occipital Pole L	-6	-96	0	1035	-863.5	0.011
PSP	7	Cerebellum Crus2 L	-6	-84	-24	162	-845.6	0.012
PSP	8	Cerebellum Crus1 L	-44	-42	-36	486	810.4	0.018
PSP	9	Frontal Superior R	24	14	60	91	-780.3	0.022
PSP	10	Frontal Pole R	28	36	-26	107	-763.2	0.025
PSP	11	Precentral L	40	-24	54	126	-752.7	0.027
PSP	12	Precentral R	-2	-22	66	214	-745.0	0.028
PSP	13	Precentral R	18	-34	54	23	-742.4	0.028
PSP	14	Inferior Temporal R	52	-30	-18	199	738.4	0.029
PSP	15	Cerebellum 9 R	12	-60	-28	226	723.1	0.034
PSP	16	Cingulum Posterior R	10	-24	42	61	-716.0	0.037
PSP	17	Cuneus L	-4	-88	42	8	-703.3	0.041
PSP	18	Cerebellum Crus1 L	-42	-76	-24	76	-702.6	0.042
PSP	19	Putamen L	-26	-6	2	16	-698.4	0.043
PSP	20	Cerebellum 6 R	42	-30	-32	14	695.1	0.043
PSP	21	Temporal Middle R	64	-40	-12	13	687.0	0.047
PSP	22	Cerebellum Crus1 R	48	-40	-36	17	683.1	0.050
svPPA	1	Temporal Pole L	-42	12	-30	5877	4882.0	0.000
svPPA	2	Temporal Pole R	42	8	-30	4789	3777.9	0.000
svPPA	3	Caudate L	-6	12	-6	1620	2194.8	0.000

svPPA	4	Insula L	-36	8	6	3057	-1502.8	0.000
svPPA	5	Frontal Medial R	-2	54	-6	236	-875.3	0.013
svPPA	6	Putamen R	22	0	-10	469	-866.7	0.013
svPPA	7	Inferior Frontal R	54	18	-6	139	-782.2	0.020
svPPA	8	Cingulum R	6	-24	26	360	-772.8	0.022
svPPA	9	Frontal Orbital R	34	18	-18	68	-755.0	0.027
svPPA	10	Frontal Pole L	0	62	12	176	-746.8	0.029
svPPA	11	Lateral Occipital L	-36	-72	14	26	714.3	0.032
svPPA	12	Parietal Operculum R	64	-24	18	90	-711.1	0.033
svPPA	13	Frontal Orbital L	-24	24	-28	66	-691.9	0.040
bFTD vs. CBS	1	Frontal Orbital R	-14	26	6	1838	1461.8	0.001
bvFTD vs. CBS	2	Parietal Superior L	-48	-46	32	3989	-1128.2	0.006
bvFTD vs. CBS	3	Cingulum Anterior R	18	30	6	559	959.8	0.009
bvFTD vs. CBS	4	Occipital Lateral L	-32	-70	6	556	-848.1	0.013
bvFTD vs. CBS	5	Postcentral R	54	-34	32	1195	-841.4	0.017
bvFTD vs. CBS	6	Precentral R	36	-12	56	517	-836.1	0.019
bvFTD vs. CBS	7	Precuneus R	-20	-66	20	127	-747.2	0.032
bvFTD vs. nfvPPA	1	Supramarginal L	-48	-48	32	474	-928.1	0.000
bvFTD vs. nfvPPA	2	Postcentral L	-26	-48	56	131	-741.6	0.000
bvFTD vs. PSP	1	Temporal Middle L	-42	-48	30	4883	-1460.1	0.000
bvFTD vs. PSP	2	Brain Stem	0	-24	-12	595	-1140.8	0.000
bvFTD vs. PSP	3	Frontal Superior R	6	26	54	1055	1094.8	0.000
bvFTD vs. PSP	4	Cingulum Anterior L	-18	20	12	749	897.7	0.005
bvFTD vs. PSP	5	Caudate R	16	26	12	540	850.9	0.009
bvFTD vs. PSP	6	Cerebellum 9 L	-12	-48	-36	328	-736.0	0.024
bvFTD vs. PSP	7	Cerebellum 9 R	6	-42	-46	130	-721.5	0.027
bvFTD vs. svPPA	1	Frontal Pole L	-38	18	2	14642	2609.9	0.000
bvFTD vs. svPPA	2	Temporal Pole L	-42	2	-30	2413	-1941.2	0.000
bvFTD vs. svPPA	3	Temporal Pole R	40	12	-30	2193	-1733.7	0.001
bvFTD vs. svPPA	4	Fusiform L	-24	-52	-18	729	-927.1	0.015
bvFTD vs. svPPA	5	Frontal Orbital R	46	18	6	527	909.4	0.016
bvFTD vs. svPPA	6	Frontal Pole L	-42	36	18	180	768.1	0.034
CBS vs. PSP	1	Thalamus L	-2	-18	-6	639	-1223.4	0.002
CBS vs. PSP	2	Cerebellum 9 L	6	-46	-28	781	-1140.4	0.003
CBS vs. PSP	3	Temporal Middle L	-66	-12	-18	220	-962.3	0.014
CBS vs. PSP	4	Cerebellum 10 L	-18	-34	-36	415	-909.4	0.017
CBS vs. PSP	5	Brain Stem	22	-34	-40	158	-851.8	0.027
CBS vs. PSP	6	Precentral R	16	-24	56	288	851.3	0.028
CBS vs. svPPA	1	Temporal Pole L	-38	8	-34	2902	-3921.0	0.000
CBS vs. svPPA	2	Temporal Pole R	36	12	-34	3402	-3004.9	0.000
CBS vs. svPPA	3	Insula L	-36	-18	20	1453	1337.8	0.000
CBS vs. svPPA	4	Cingulum Posterior	18	-12	36	2732	1318.6	0.000
CBS vs. svPPA	5	Frontal Pole L	-20	54	18	843	901.0	0.014

CBS vs. svPPA	6	Frontal Pole R	34	48	6	105	880.4	0.016
nfvPPA vs. PSP	1	Cerebellum 8 L	22	-36	-42	2569	-1011.4	0.007
nfvPPA vs. svPPA	1	Temporal Pole R	42	12	-28	3586	-2411.1	0.000
nfvPPA vs. svPPA	2	Temporal Pole L	-42	12	-28	1626	-2046.4	0.000
nfvPPA vs. svPPA	3	Frontal Pole L	-38	6	12	6194	1549.4	0.000
nfvPPA vs. svPPA	4	Frontal Orbital L	-26	24	-18	323	795.7	0.022
PSP vs. svPPA	1	Temporal Pole R	46	6	-28	2905	-3660.7	0.000
PSP vs. svPPA	2	Temporal Pole L	-42	8	-30	1367	-2316.5	0.000
PSP vs. svPPA	3	Insula L	-48	-4	6	5552	2210.3	0.000
PSP vs. svPPA	4	Brain Stem	-12	-24	-30	9541	1882.6	0.000
PSP vs. svPPA	5	Precentral R	40	-18	42	595	-1191.0	0.001
PSP vs. svPPA	6	Precentral R	36	-4	48	247	-974.8	0.007
PSP vs. svPPA	7	Paracingulate R	-6	54	-6	1220	935.1	0.009
PSP vs. svPPA	8	Precentral L	-2	-30	60	471	-932.1	0.009
PSP vs. svPPA	9	Occipital Pole L	10	-84	2	776	-882.9	0.014
PSP vs. svPPA	10	Thalamus R	12	-12	6	119	-865.9	0.018
**Fractional amplitude of low-frequency fluctuations**								
bvFTD	1	Frontal Pole	0	8	48	107551	-5925.5	0.000
bvFTD	2	Temporal Pole	68	-50	-14	251	-1112.1	0.043
bvFTD	3	Cerebellum 4_5 R	16	-48	-16	40	-1064.1	0.050
CBS	1	Occipital Lateral L	-38	-72	36	467	-1261.3	0.029
CBS	2	Frontal Pole L	-2	36	54	994	-1257.2	0.029
CBS	3	Precentral L	-42	2	54	177	-1214.7	0.034
CBS	4	Temporal Pole L	-60	-60	30	335	-1181.1	0.040
CBS	5	Calcarine L	-8	-60	12	26	-1093.2	0.047
CBS	6	Frontal Pole R	16	36	56	9	-1072.3	0.047
CBS	7	Temporal Pole L	-66	-48	2	6	-1070.3	0.049
CBS	8	Precentral L	-36	6	38	2	-1067.0	0.049
svPPA	1	Insula L	-36	8	6	207	-1020.0	0.021
bvFTD vs. svPPA	1	Frontal Pole R	28	14	48	53257	-2797.5	0.010
PSP vs. svPPA	1	Thalamus R	10	-4	6	249	-1342.0	0.035

bvFTD: behavioral variant, CBS: cortico-basal syndrome, L: left, nfvPPA: nonfluent variant primary progressive aphasia, p_{FWE}: probability corrected by family-wise error, PSP: progressive supranuclear palsy, R: right, size: number of voxels, svPPA: semantic variant primary progressive aphasia, TFCE: threshold-free cluster enhancement.

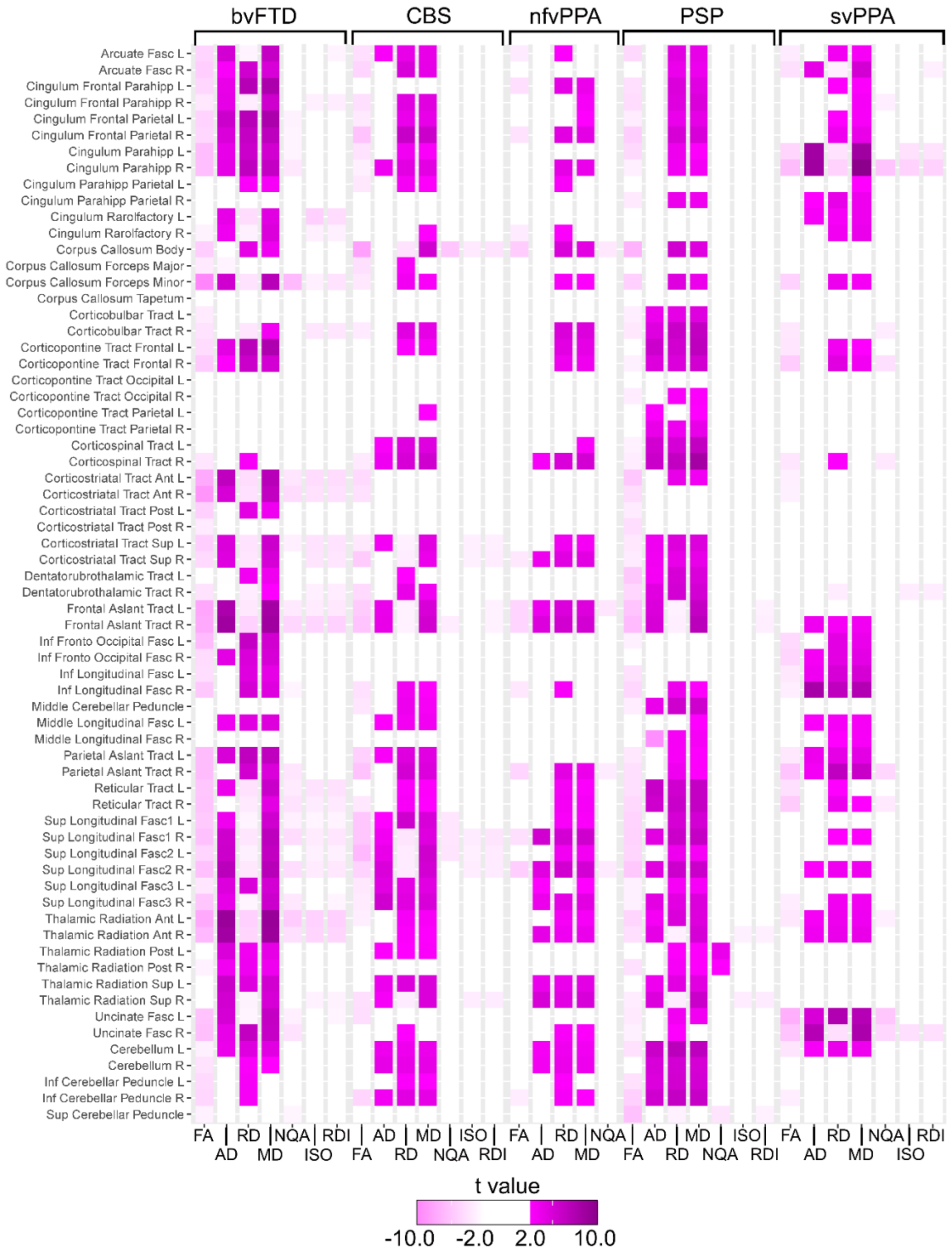

Supplementary Figure 5. Raw structural connectivity in five variants of frontotemporal dementia. Every variant was compared with a sample of healthy controls matched in sex, age, and years of education. The multiple comparisons were controlled by FDR ($p_{FDR} < 0.05$). AD: axial

diffusivity, Ant: anterior, bvFTD: behavioral variant, CBS: cortico-basal syndrome, FA: fractional anisotropy, Fasc: Fasciculus, Inf: inferior, ISO: isotropic diffusion component derive, L: left, MD: mean diffusivity, NQA: normalized quantitative anisotropy, nfvPPA: nonfluent variant primary progressive aphasia, Post: posterior, PSP: progressive supranuclear palsy, R: right, RD: radial diffusivity, RDI: restricted diffusion imaging, Sup: Superior, svPPA: semantic variant primary progressive aphasia, TFCE: threshold-free cluster enhancement.

References

Boxer, A. L., Geschwind, M. D., Belfor, N., Gorno-Tempini, M. L., Schauer, G. F., Miller, B. L., . . . Rosen, H. J. (2006). Patterns of brain atrophy that differentiate corticobasal degeneration syndrome from progressive supranuclear palsy. *Archives of Neurology, 63*, 81-86. doi:10.1001/archneur.63.1.81

Cohen, J. (1988). Statistical Power Analysis for the Behavioral Sciences. 412. doi:https://doi.org/10.4324/9780203771587

Glasser, M. F., Coalson, T. S., Robinson, E. C., Hacker, C. D., Harwell, J., Yacoub, E., . . . Van Essen, D. C. (2016). A multi-modal parcellation of human cerebral cortex. *Nature, 536*(7615), 171-178. doi:10.1038/nature18933

Lu, P. H., Mendez, M. F., Lee, G. J., Leow, A. D., Lee, H. W., Shapira, J., . . . Knopman, D. S. (2013). Patterns of Brain Atrophy in Clinical Variants of Frontotemporal Lobar Degeneration. *Dementia and Geriatric Cognitive Disorders, 35*, 34-50. doi:10.1159/000345523

Seeley, W. W., Crawford, R. K., Zhou, J., Miller, B. L., & Greicius, M. D. (2009). Neurodegenerative Diseases Target Large-Scale Human Brain Networks. *Neuron, 62*, 42-52. doi:10.1016/j.neuron.2009.03.024

Smith, S. M., & Nichols, T. E. (2009). Threshold-free cluster enhancement: Addressing problems of smoothing, threshold dependence and localisation in cluster inference. *NeuroImage, 44*, 83-98. doi:10.1016/j.neuroimage.2008.03.061

Venkatraman, E. S. (2000). A permutation test to compare receiver operating characteristic curves. *Biometrics, 56*, 1134-1138.

Whitwell, J., Jack, C. R., Boeve, B. F., Parisi, J. E., Ahlskog, J. E., Drubach, D. A., . . . Josephs, K. A. (2010). Imaging correlates of pathology in corticobasal syndrome. *Neurology, 75*, 1879-1887.

Whitwell, J., Przybelski, S. A., Weigand, S. D., Ivnik, R. J., Vemuri, P., Gunter, J. L., . . . Josephs, K. A. (2009). Distinct anatomical subtypes of the behavioural variant of frontotemporal dementia : a cluster analysis study. *Brain, 132*, 2932-2946. doi:10.1093/brain/awp232

Wilcox, R. R. (2017). Introduction to Robust Estimation and Hypothesis Testing. 777.
